# The utility of DNA metabarcoding for studying the response of arthropod diversity and composition to land-use change in the tropics

**DOI:** 10.1038/srep24965

**Published:** 2016-04-26

**Authors:** Kingsly Chuo Beng, Kyle W. Tomlinson, Xian Hui Shen, Yann Surget-Groba, Alice C. Hughes, Richard T. Corlett, J. W. Ferry Slik

**Affiliations:** 1 Center for Integrative Conservation, Xishuangbanna Tropical Botanical Garden, Chinese Academy of Sciences, Menglun, Mengla, Yunnan, 666303, China; 2 University of Chinese Academy of Sciences, No. 19A Yuquan Road, Beijing 100049, China; 3 Institut des Sciences de la Forêt Tempérée, Université du Québec en Outaouais, 58, Rue Principale, Ripon, Québec J0V 1V0, Canada; 4 Quebec Centre for Biodiversity Science, McGill University, 1205 Dr. Penfield Avenue, Montreal, Quebec, H3A 1B1, Canada; 5 Faculty of Science, Universiti Brunei Darussalam, Jln. Tungku Link, Gadong, BE1410, Brunei Darussalam

## Abstract

Metabarcoding potentially offers a rapid and cheap method of monitoring biodiversity, but real-world applications are few. We investigated its utility in studying patterns of litter arthropod diversity and composition in the tropics. We collected litter arthropods from 35 matched forest-plantation sites across Xishuangbanna, southwestern China. A new primer combination and the MiSeq platform were used to amplify and sequence a wide variety of litter arthropods using simulated and real-world communities. Quality filtered reads were clustered into 3,624 MOTUs at ≥97% similarity and the taxonomy of each MOTU was predicted. We compared diversity and compositional differences between forests and plantations (rubber and tea) for all MOTUs and for eight arthropod groups. We obtained ~100% detection rate after *in silico* sequencing six mock communities with known arthropod composition. Ordination showed that rubber, tea and forest communities formed distinct clusters. α-diversity declined significantly between forests and adjacent plantations for more arthropod groups in rubber than tea, and diversity of order Orthoptera increased significantly in tea. Turnover was higher in forests than plantations, but patterns differed among groups. Metabarcoding is useful for quantifying diversity patterns of arthropods under different land-uses and the MiSeq platform is effective for arthropod metabarcoding in the tropics.

Following anthropogenic pressure, global ecosystems have undergone more severe and rapid changes during the past few decades than in any other period in Earth history^1^,^2^. These changes constitute the greatest environmental challenges we face today, especially anthropogenic climate change, biodiversity loss and biological invasions^2^. The massive and ongoing environmental degradation in the Anthropocene emphasizes the need for efficient and fast methods of quantifying and monitoring the spatial and temporal dynamics of biodiversity^3^,^4^. This is especially true for megadiverse, highly cryptic and relatively small-sized organisms, where traditional methods of biodiversity assessment are relatively slow, expensive and time consuming^5^,^6^. Metabarcoding, the combination of DNA taxonomy and high-throughput sequencing, is a promising tool for the rapid assessment and monitoring of biodiversity in mixed, bulk samples^7^. Metabarcoding has been successfully applied to taxa that are difficult to assess with traditional methods, including protists^8^,^9^, fungi^10^,^11^, bacteria^12^,^13^, nematodes^14^,^15^, and annelids^16^,^17^. These studies not only reveal the hidden diversity within these taxa by allowing a more complete discovery of taxonomic diversity when compared to traditional methods, but also reliably quantify patterns of diversity and assemblage composition along environmental gradients. This information is critical for timely biodiversity monitoring, conservation management, land-use planning, and environmental impact assessment.

Although arthropods constitute the most abundant and diverse non-microbial organisms on Earth^18^,^19^,^20^, comprehensive information on large-scale patterns of richness, endemism and biogeography are lacking, especially in the tropics^21^. Arthropods are hyperdiverse, highly cryptic and relatively small-sized organisms and their low representation in biodiversity databases is due to the high cost (in terms of money, time and labor) associated with sorting and identifying samples from large-scale inventories^5^,^6^. Cheap, quick and efficient monitoring methods are therefore urgently needed to address this impediment. Metabarcoding has been successfully used to assess patterns of arthropod diversity and assemblage composition, and this technique has proven to be faster, cheaper, and more comprehensive than traditional morphological taxonomy^5^,^22^. However, previous metabarcoding studies on non-microbial organisms have mostly used Roche 454 platforms^5^,^22^,^23^, and to our knowledge only one study has used the MiSeq platform for Malaise trapped arthropod samples^24^. Since the Miseq platform is more cost-efficient than Roche 454 for metabarcoding^6^,^22^,^23^, and can produce up to ca. 15 times the number of reads produced by a Roche 454 FLX Titanium run^22^,^25^,^26^, it may represent a promising alternative for efficient biodiversity assessment and monitoring.

One limitation of metabarcoding is the efficiency of assigning taxonomy to molecular operational taxonomic units (MOTUs). Though the percentage of MOTUs assigned to order level is usually high, this is not the case for assignments at a lower taxonomic level (e.g. for insects; Order [96–99%], Family [17–37%], Genus [16–36%] and Species [16–35%]^6^). This problem is not due to the metabarcoding pipeline used, but rather to the lack of comprehensive and taxonomically reliable barcode databases for most taxa^6^,^27^. To enhance the utility of metabarcoding for large-scale and long-term biodiversity monitoring, it is important to increase the identification and standard barcoding of species, especially in the highly diverse tropics. Using a new primer combination targeting a 400 bp fragment of the COI gene and Illumina high-throughput sequencing, we seek to show the effectiveness of the Miseq platform for metabarcoding a wide variety of tropical arthropods. While most previous studies focused on general diversity patterns without inferring group-specific (e.g. order) differences^5^,^6^, we tried to detect finer patterns by assigning MOTUs to different classes and orders and analyzing diversity patterns for each order/class separately. To investigate the utility of DNA metabarcoding in a study of patterns of litter arthropod diversity across land-use types in Xishuangbanna, we address the following questions:

1. Can the MiSeq platform be used effectively for arthropod metabarcoding in the tropics?
2. Do clustered MOTUs indicate significant community differentiation across land-use types and measured environmental gradients?
3. Which arthropod groups show significant changes across land-uses and deserve further attention?

## Results

### *In silico* amplification, sequencing, OTU clustering and mock community validation

To test the efficiency of the MiSeq platform for arthropod metabarcoding, we verified whether the method worked successfully with mock communities. Firstly, we tested whether combining the primers MHemF^28^ and dgHCO2198^29^ could successfully amplify (*in silico* PCR) the COI gene for arthropod sequences using 37 reference arthropod sequences combined in 6 different libraries with different numbers of reads. In PCR simulations, the primer set amplified the COI gene of all 37 arthropod species in accordance with each library’s input specifications (Table 1). The quality filtered reads of all mock communities except one (library 2) were faithfully clustered into the correct number of OTUs (Table 1). All OTUs had perfect matching with the reference sequences from which they were generated (Table S2).

### Primer efficiency, Illumina sequencing and MOTU recovery on field samples

The primer set MHemF and dgHCO2198 showed high amplification success for a wide range of arthropod species and bulk arthropod samples (Fig. S1, Supporting Information). The total number of reads that passed the default Illumina filtering step was 2,628,704 96 (37,553 [mean] ± 10,373 [s.d.] per site) and the mean length of each read was 439 bp (range: 368–454 bp). After custom quality filtering (primers and barcodes stripped, and read length truncated), 2,573,479 reads (36,764 [mean] ± 10,361 [s.d.] per site) were retained (Table S3, Supporting Information). The dereplication step yielded 1,832,637 sequences (unique sequence with abundance >2) and 1,728,491 unique singletons. The 1,728,491 unique singletons were discarded while the 1,832,637 sequences were clustered into 3,624 MOTUs at 97% similarity. During MOTU clustering, 865 chimeras were detected and discarded.

### Taxonomic identification of MOTUs from field samples

Out of the 3,624 MOTUs, UTAX predicted taxonomy for 3,588 MOTUs (99.0% prediction) while USEARCH gave top hits (≥75% identity to reference database) for 3,249 MOTUs (94.6% prediction). UTAX and USEARCH taxonomic assignments showed high similarity across arthropod groups. Out of the 261 MOTUs assigned to Hymenoptera by UTAX, 253 were also assigned by USEARCH (97% correspondence). Details of the comparison between the two taxonomic assignment algorithms (UTAX and USEARCH) for MOTUs assigned to Hymenoptera can be found in Table S4 (Supporting Information).

### Differences in species diversity and composition across land-use types

**Figure 1:**
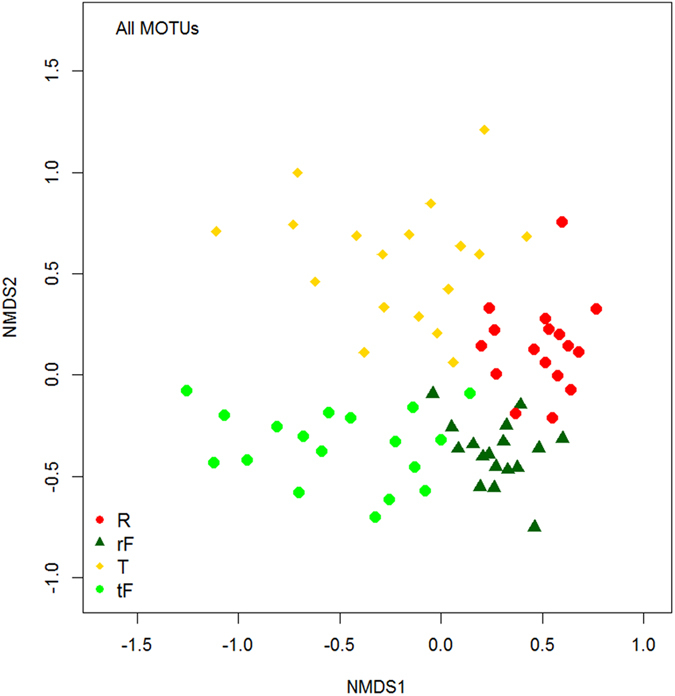
Ordination (nMDS) plot illustrating the similarities and differences in MOTU composition across four land-use types. R = rubber, T = tea, while rF and tF = forest matched with rubber and tea, respectively.

Ordination plots showed that the habitat types have clearly distinct MOTU assemblages, with each habitat type forming a separate group (Fig. 1). Interestingly, forests associated with rubber were also distinct from forests associated with tea. The first axis of the ordination plot (NMDS1) was highly correlated with the horizontal distance between land-use pairs [positive], and elevation [negative], whereas NMDS2 was highly correlated with slope [negative], litter thickness [negative], canopy openness [negative], and litter chemistry (total carbon [negative], total nitrogen [positive], total phosphorus [positive], coarse fiber content [negative] and lignin content [negative]) (Table 2; Fig. S2, Supporting Information). Similar patterns of community composition were also observed across individual arthropod groups, with the exception of Chilopoda which had almost zero stress due to insufficient data (Figs S3–S9, Supporting Information).

**Figure 2:**
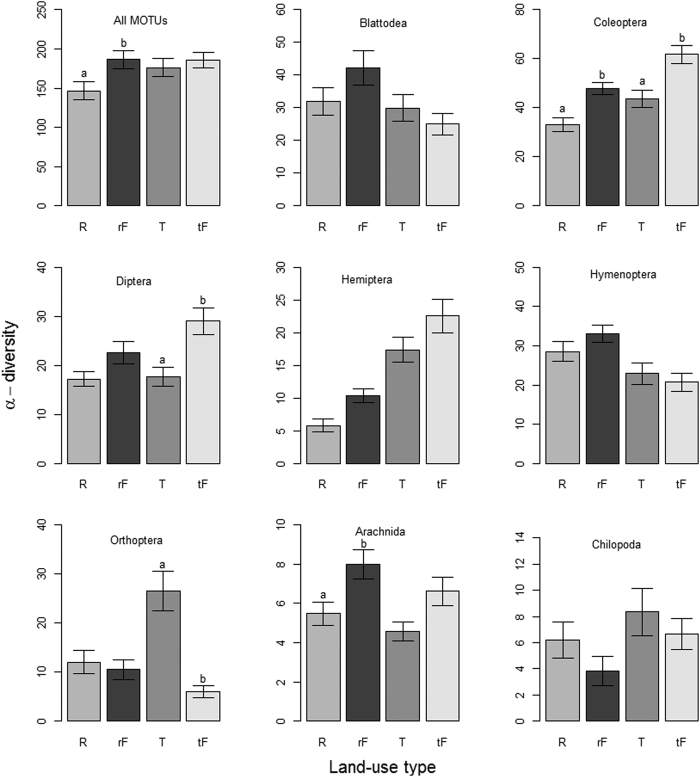
Comparison of α-diversity (mean ± s.e.m.) in matched forest-plantation sites for all MOTUs and eight arthropod orders. All tests are based on Kruskal–Wallis followed by Mann–Whitney post-hoc comparisons with Bonferroni correction. Significant differences between pairs [R vs. rF and T vs tF] are indicated with different lowercase characters (a, b). R = rubber, T = tea, while rF and tF = forest matched with rubber and tea, respectively.

Local MOTU richness slightly increased with sampling size (total number of reads per site) but this increase did not affect overall α-diversity patterns (Fig. S10). The number of observed MOTUs correlated linearly with the number of rarefied MOTUs (Fig. S10). Pairwise differences in MOTU (α- and β-) diversity varied considerably between land use pairs and across arthropod groups. Overall, MOTU α-diversity was significantly higher in the forest sites adjacent to rubber (hereafter rubber-forest) than in rubber, but not significantly higher in forests adjacent to tea (hereafter tea-forest) than tea (Fig. 2). Pairwise alpha diversity patterns of the main arthropod groups also differed across land use types. Coleoptera richness was lower in tea and rubber plantations than adjacent forests, and richness of All MOTUs and Arachnida, was lower in rubber than in rubber-forests, Diptera richness was lower in tea than in tea-forests whereas Orthoptera richness was higher in tea than tea-forests. Blattodea, Hemiptera, Hymenoptera and Chilopoda richness did not differ across land-uses. Three-way (forest and rubber, forest and tea, and rubber and tea) alpha diversity patterns showed that All MOTUs, Coleoptera, and Hemiptera richness was significantly higher in forests than in rubber (Table 3). Arachnida, Coleoptera, and Diptera richness was significantly higher in forests than in tea. Orthoptera richness was significantly higher in tea than in forests. Coleoptera, Hemiptera and Orthoptera richness was significantly higher in tea than in rubber. We did not detect any substantial differences in diversity between terraced and non-terraced tea, and between monoculture and polyculture tea (Table S5, Supporting Information).

**Figure 3:**
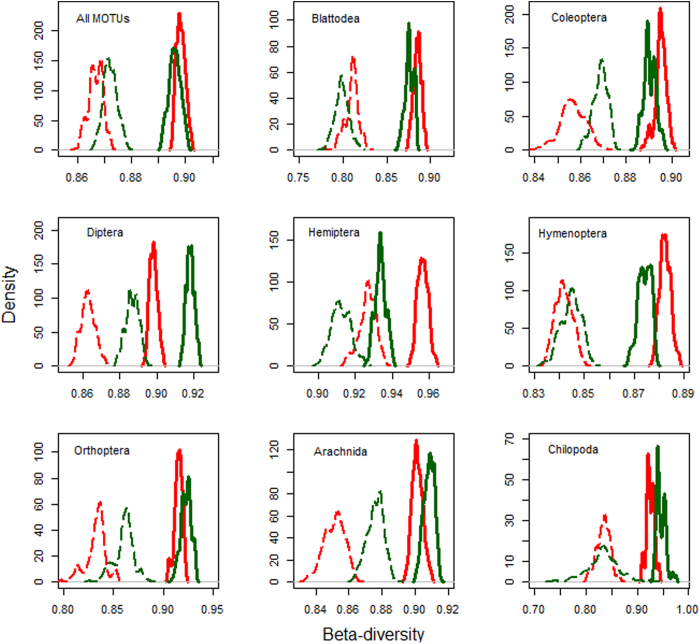
Total β-diversity (smooth lines) and turnover (broken lines) for rubber-forests [green lines] versus rubber [red lines] sites. These were computed using 1000 bootstrap samples of 15 sites from each land use type. Significant differences between pairs are detected when the peaks of the density plots do not overlap with each other.

**Figure 4:**
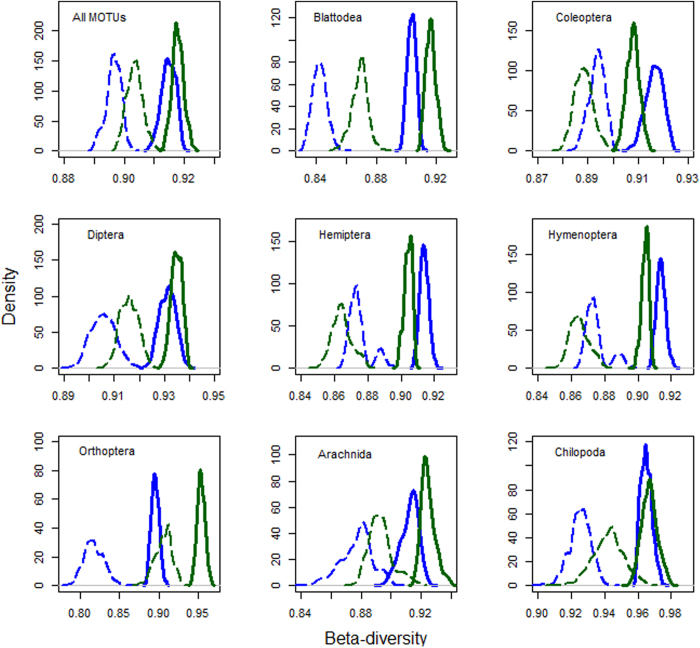
Total β-diversity (smooth lines) and turnover (broken lines) for tea-forests [green lines] versus tea [blue lines] sites. These were computed using 1000 bootstrap samples of 15 sites from each land use type. Significant differences between pairs are detected when the peaks of the density plots do not overlap with each other.

We detected higher levels of turnover between sites (species replacement by new species not found elsewhere) in rubber-forests than in rubber for All MOTUs, Coleoptera, Diptera, Hymenoptera, Orthoptera and Arachnida (Fig. 3), with rubber characterized by higher nestedness (gain and loss of species also found elsewhere). Similarly, higher levels of turnover were detected in tea-forests than in tea for All MOTUs, Blattodea, Diptera, Orthoptera, Arachnida and Chilopoda (Fig. 4).

When considering species turnover against geographic distance between sampling locations, patterns of turnover with distance were highly significant for tea-forests and tea than for rubber-forests and rubber across arthropod groups (Table 4). There were no significant effects of distance on arthropod turnover in rubber, but distance did significantly influence Coleoptera turnover in rubber-forests. Arthropod species turnover with distance was significant for both tea-forests and tea for All MOTUs, Diptera, Hemiptera, Hymenoptera, and Arachnida, significant for only tea-forests for Coleoptera, and significant only for tea for Orthoptera (Table 4).

## Discussion

Previous metabarcoding studies on non-microbial organisms have mostly used Roche 454 platforms^5^,^6^,^23^, and to our knowledge only one study has used the MiSeq platform for Malaise trapped arthropod samples^24^. The Miseq platform is more cost-efficient than Roche 454 for metabarcoding^6^,^22^,^23^ and can produce up to ca. 15 times the number of reads produced by a Roche 454 FLX Titanium run^22^,^25^,^26^. We used a new primer combination targeting a 400 bp fragment of the COI gene and Illumina high-throughput sequencing to demonstrate the effectiveness of the Miseq platform for metabarcoding a wide variety of tropical arthropods. While this fragment is shorter than the regular barcode fragment (ca. 650 bp), it still allowed a perfect identification of the samples in the mock communities we simulated.

Arthropods constitute the most abundant and diverse non-microbial organisms on Earth, but comprehensive information on large-scale patterns of richness, endemism and biogeography are lacking, especially in the tropics^18^,^19^,^20^,^21^. The low representation of arthropods in biodiversity databases is due to the high cost (in terms of money, time and labor) associated with sorting and identifying samples from large-scale inventories^5^,^6^. A cheap and efficient monitoring method such as presented in this study will greatly help to address this impediment.

A major limitation of this approach is the efficiency of the MOTU taxonomic assignment. While most previous studies focused on general diversity patterns without assigning taxonomy to MOTUs^5^,^6^,^23^, we detected finer patterns by assigning MOTUs to different classes and orders and analyzed diversity patterns for each order/class separately. Though the taxonomy assignment at this high level is very efficient (97 to 99% depending on the method used), it is not the case for assignments at a lower taxonomic level (from 64% in Orthopteras to only 21% for Chilopoda, Table S6). This is not a problem due to the metabarcoding pipeline we used, but rather to the lack of good reference barcode sequences for tropical arthropods. Before being able to use metabarcoding for detailed biodiversity monitoring, we stress the importance of increasing the identification and standard barcoding of arthropod species in the highly diverse tropics.

The four land-use types considered had distinct arthropod communities, showing that each land-use class supports a unique arthropod assemblage. The strong differences in community structure found between tea and adjacent forests and between rubber and adjacent forests suggest that arthropods are rather sensitive to land-use change^30^,^31^,^32^.

Furthermore, species turnover between sites in plantations mainly represented species losses and gains with few new species added (nestedness), while species turnover between sites in forests was dominated by addition of new species not found anywhere else (turnover after accounting for nestedness). Although overall compositional diversity remained high in plantations, our results do point to a homogenizing compositional trend in plantation landscapes^33^.

Arthropod compositional patterns varied considerably across land-use pairs and arthropod groups. We found strong correlations between environmental gradients and species compositional changes across land-use types, suggesting that the interactions among land-use change, environmental heterogeneity and species life-history might be driving differences in β-diversity^34^,^35^. Our study confirms the importance of monitoring and understanding changes in species composition (rather than just species numbers), supporting similar pleas from related studies^36^,^37^.

Diversity (α-) was generally high in native tropical forests and lower in adjacent agricultural plantations, with greater changes detected between rubber and forest than between forest and tea. Our findings are consistent with reports that landscape modification has negative effects on biodiversity, and these effects vary across functional guilds^31^,^38^,^39^. The lower numbers of species in monoculture plantations (especially rubber) corroborate previous findings that clearance of natural forests and subsequent conversion to agriculture leads to loss of specialist forest-dwelling species and colonization by generalist landscape species that can tolerate relatively harsh conditions (e.g. high temperature, high solar radiation, pesticide application) within agricultural landscapes^36^,^37^.

The effects of land-use change on arthropod diversity were mostly negative, but were also positive or non-existent for some arthropod groups. This is consistent with previous reports that taxa differ in their responses to land-use change^31^,^38^,^39^. One positive effect of forest conversion was the increase in Orthoptera richness in tea relative to tea-forest. A possible explanation may be the increase in open environment species like grasshoppers, which largely colonize and proliferate in tea. The most negatively affected arthropod order was Coleoptera, which showed significantly higher α-diversity in forests than in neighboring plantations (rubber and tea), higher turnover in rubber-forest than in rubber and significant correlations between turnover and geographic distances in forests but not in neighboring plantations (rubber and tea). This confirms the interest in using this group of arthropods to monitor changes in biodiversity and forest degradation^40^,^41^,^42^. For arthropod order Hymenotera, species turnover between sites was higher in rubber-forests than in rubber but lower in tea-forests than in tea, and significant correlations between turnover and geographic distances were detected in tea-forests and in tea plantations but not in rubber-forests and rubber plantations. These findings can be attributed to the higher occurrence of ants (an important component of litter Hymenopterans) in disturbed than in undisturbed habitats^31^, the positive correlation between ant species richness and temperature^43^, and the negative correlation between ant species richness and disturbance^43^. A similar study comparing ant species richness and composition in forest, agroforestry rubber, monoculture rubber and oil palm plantations in Indonesia found that agricultural land-use alters species composition but not species richness of ants^44^.

## Materials and Methods

### Study site

**Figure 5:**
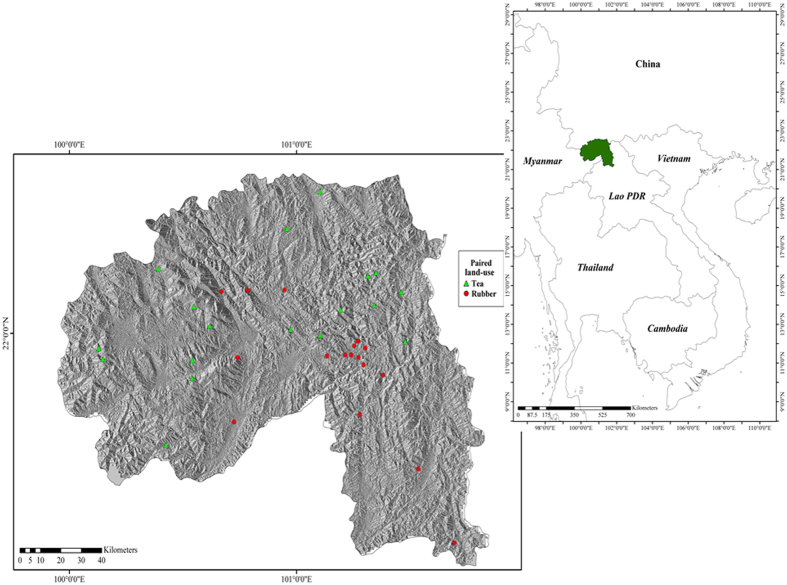
Location of Xishuangbanna (XSBN) in Yunnan province, China and in Southeast Asia (right; green) and paired sample sites where arthropods were collected (left). The green triangles represent forest-tea paired sites and the red circles represent forest-rubber paired sites. The regional map (China, Cambodia, Lao PDR, Myanmar, Thailand and Vietnam) was generated using the TM_world_borders_3 dataset freely available from the thematic mapping website (http://thematicmapping.org/downloads/world_borders.php). The prefecture (XSBN) map was generated using the freely available level three (provincial) maps from Divagis spatial data (http://www.diva-gis.org/; Hijmans, R. J., L. Guarino, C. Bussink, P. Mathur, M. Cruz, I. Barrentes, and E. Rojas. 2004. DIVA-GIS. Version. 5.0. A geographic information system for the analysis of species distribution data). Background data shows the slope aspect, generated from the Shuttle Radar Topography Mission (SRTM) 1 Arc-Second Global using the “aspect” tool in ArcMap 10.1 (ESRI 2015. ArcGIS Desktop: Release 10.1. Redlands, CA: Environmental Systems Research Institute; http://desktop.arcgis.com/en/arcmap/). Points were downloaded from a hand-held GPS (GPSMAP® 62s | Garmin) before being digitized and uploaded as a shapefile.

The study was conducted within the Xishuangbanna (XSBN) Dai Autonomous Prefecture (21°08′N-22°36′N, 99°56′E-101°50′E) of Yunnan Province, SW China (Fig. 5). XSBN lies on the northern edge of tropical Southeast Asia^45^ within the Indo-Burma biodiversity hotspot^46^. The topography is mountainous, with altitudes ranging from 542–2415 m above sea level. XSBN experiences a tropical monsoon climate with a distinct hot, rainy (May-October) and cool, dry season (November-April). Although XSBN’s climate is generally warm and moist, both temperature and rainfall vary considerably over the prefecture^47^. Using high resolution geospatial monthly climate data (1960–2000), four bioclimatic zones were identified within XSBN^47^, ranging from hot/moist climates at low elevations (<600 m a.s.l.) to warm temperate/mesic climates at high elevations (>2,000 m a.s.l.). Mean annual temperature varies from 14.9 °C (>2000 m a.s.l.) to 23.4 °C (<600 m a.s.l.) while mean annual rainfall varies from 1,624 mm (>2,000 m a.s.l.) to 1,222 mm (<600 m a.s.l.).

Although XSBN represents only 0.2% (1.9 million ha) of China’s total area, the region supports an estimated 16% of China’s total higher plants^48^ and substantial arthropod diversity^5^,^6^,^22^,^23^. The region’s land cover is substantially fragmented with the most prominent changes caused by forest conversion to agriculture, especially plantations of rubber (*Hevea brasiliensis*), an exotic crop, in the lowlands, and tea (*Camellia sinensis*), an indigenous crop, at higher elevations. The area covered by rubber plantations has increased rapidly from 87,000 ha (4.6% of XSBN’s total area) in 1992 to 424,000 ha (22.3% of XSBN’s total area) in 2010, at the expense of tropical forests^49^. Tea plantations have been part of the landscape for a longer time, but the total land area under tea cultivation in XSBN is unknown. Details of the different land-use types and their characteristics and the variables measured at each site can be found as Supplementary Text.

### Sample collection

Bulk litter arthropod samples were collected from 35 matched forest-plantation sites across XSBN. Pairs were selected to be as similar as possible and spatially close to minimize confounding differences in environmental conditions. Land-use types studied included native vegetation (forest), rubber plantations (rubber) and tea plantations (tea). In each site, nine leaf-litter samples (placed 10 m apart; one in the middle and two each in north, east, west and south directions) were collected by placing 1 × 1 m PVC frames on the ground (Fig. S11, Supporting Information). All leaf litter and loose humus from within the frame area were collected into a large polythene bag and sieved through a wire mesh (0.8 cm × 0.8 cm) to remove larger leaf-litter materials. The resulting ‘siftate’ was transported to the laboratory in polythene bags, where it was immediately transferred into mesh bags. The mesh bags and their contents were subsequently suspended inside Winkler bags containing bottles with 96% alcohol at the bottom. The Winkler bags were left to dry for three days in a room with air conditioning to make arthropods leave the litter in search of moisture. The suspended ‘siftate’ was gently mixed during incubation to increase the activity of arthropods and their chances of dropping into the collection bottle^50^,^51^,^52^.

### Sample preparation and DNA extraction

Arthropod samples from each site were prepared separately by pouring the contents of the collection bottle into a clean sterile petri dish. Each petri dish was placed under a stereomicroscope and sterile forceps were used to pick out all visible arthropods. The arthropods were stored in clean sterile bottles containing 96% ethanol at room temperature until DNA extraction. In order to keep the final DNA quantity similar across individual arthropods, we used two legs from all individuals with body length equal to or greater than 5 mm and whole bodies of everything smaller. These samples were subsequently freeze-dried using liquid Nitrogen, ground and homogenized using a mortar and pestle. Genomic DNA was extracted using the DNeasy Tissue Kit (QIAGEN; Hilden, Germany; protocol for animal tissues) according to the manufacturer’s instructions.

### Primer test and pipeline validation with mock arthropod communities

Since the COI barcode fragment is too long for sequencing on the MiSeq platform, we used a new primer combination (MhemF^28^ and dgHCO2198^29^) to amplify a fragment of ca. 400 bp. We first tested *in-vitro* PCR efficiency on a wide range of arthropods (Chilopoda, Araneae, Hymenoptera, Blattodea, Mantodea, Coleoptera, Orthoptera, Lepidoptera, and Hemiptera). PCR was carried out in a total volume of 50 μL using 10 ng DNA, 5.0 μL 10 ×  PCR buffer, 0.5 mM dNTPs, 2.5 U Platinum Taq (TaKaRa Biosystems, Ohtsu, Shiga, Japan) 0.5 μL of each of forward and reverse primers. PCR cycling conditions were 94 °C for 3 min, 5 cycles of 94 °C for 30 s; 45 °C for 20 s; 72 °C for 30 s; then 20 cycles of 94 °C for 20 s; 55 °C for 20 s; 72 °C for 30 s and finally 72 °C for 5 min. PCR products were size-verified by gel electrophoresis. Then, since the UPARSE bioinformatics pipeline has only been validated for microbes (bacteria and fungi)^53^, we validated the pipeline by simulating reads from mock arthropod communities using Grinder v.0.5.3^54^ and processing the simulated data with USEARCH v.8.1^53^. We downloaded a COI reference database of 3,306,508 arthropod sequences from the Barcode of Life database (BOLD^55^). We then used a subset of the reference (high quality sequences covering a wide range of arthropod groups from across the entire arthropod phylogeny (Table S1, Supporting Information)) to generate six mock communities. Three mock communities were constructed using 37 reference sequences, with each community assigned a unique multiplex identifier (MID). *In silico* PCR was simulated from the reference sequences using Illumina sequencing, requesting 200,000 (library 1), 400,000 (library 2) and 600,000 (library 3) reads with 300 bp length, Phred quality scores (10–40) and Illumina errors using the 4th degree polynome 3e-3 + 3.3e-8 × i^4^56^ model. To evaluate the effects of local richness and read abundance on pipeline performance, the remaining three mock communities were generated using 25 of the 37 unique reference sequences, with similar simulations (300 bp length, Phred scores and Illumina error model). The resulting fastq output files were passed to USEARCH v8.1.1861 bioinformatics pipeline for downstream analysis^53^.

### Data preparation

Primer plus barcode sequences were stripped using python scripts (*fastq_strip_barcode_relabel2.py*) in USEARCH v8.1.1861. Forward and reverse fastq files were merged (*-fastq_mergepairs*), quality filtered (-*fastq_filter*), and reads less than 250 bp in length (-*fastq_minlen*) were discarded^53^.

### Mock community Operational Taxonomic Unit (OTU) recovery

OTU picking for each library was performed using the USEARCH v8.1.1861 Illumina paired reads pipeline^53^ as follows; Reads were dereplicated, sorted by abundance and singletons were removed. The remaining reads were clustered into OTUs at a minimum similarity of 97%^57^. This clustering step also discards reads that have chimeric models built from more abundant sequences. Finally, the UPARSE-REF algorithm was used to validate the mock community sequencing experiment^53^. This algorithm is useful for understanding what picked OTUs actually represent (e.g. whether the OTUs represent expected species, contaminants, read errors, or chimeras).

### Library construction and sequencing

Amplification was carried out as described above, and PCR products were quantified using Qubit 2.0 Fluorometer (dsDNA HS Assay, Life Technologies). The amplified DNA was ligated with two standard adaptors that allow the final product to bind or hybridize to short oligos on the surface of the Illumina flow cell. These adaptors included 7 bp unique index sequences to enable multiplexing of more than one sample in the same run. A total of 36 barcode-primer combinations were synthesized and used in two runs (since we had a total of 70 samples). Purified PCR products (with adaptor and barcode sequences) from each run were pooled accordingly to form two separate sequencing libraries. Each library was quantified using Qubit 2.0. Fluorometer to determine an appropriate volume of library for sequencing. For each library, amplification was carried out in a total volume of 50 μL using 20 ng DNA, 5.0 μL 10 ×  PCR buffer, 0.5 mM dNTPs, 2.5 U Platinum Taq, and 0.5 μL of each of forward and reverse primers. PCR cycling conditions were 95 °C for 30 s, 5 cycles of 95 °C for 15 s; 55 °C for 15 s; 72 °C for 30 s and finally 72 °C for 5 min. Sequencing was performed with 2 × 300 cycles using the MiSeq Reagent Kit v3 (Illumina, Inc., 2015) as per manufacturer’s instructions. PCR amplifications, barcode design, library preparation and sequencing were done at Sangon Biotech (Shanghai) Co., Ltd.

### OTU taxonomic prediction for field samples

The taxonomies of clustered OTUs from field samples were predicted using two taxonomy prediction algorithms (UTAX and USEARCH) and the resulting taxonomic identities of each OTU were compared. UTAX is a k-mer based method which looks for words in common between the query sequence and reference sequences with known taxonomy. A score calculated from word counts is used to estimate a confidence value for each taxonomic level^53^. USEARCH searches a reference database for high-identity hits to one or more reference sequences (“targets”) using word counts to prioritize the database search. Target sequences are compared to the query in order of decreasing unique word count^53^. For UTAX, we downloaded and used a database of 840,074 Animalia COI sequences and an associated taxconfs file from the previous version of USEARCH v8.1.1831^53^. For USEARCH, we downloaded and used a database of 3,306,508 Arthropoda COI sequences from the Barcode of Life Database (BOLD^55^). We used a recommended nucleotide top hit identity cutoff of 75% for which USEARCH is effective^57^.

### Diversity and assemblage composition analysis

Since the total number of reads used to pick Molecular Operational Taxonomic Units (MOTUs) varies considerably across sampled sites, a risk of local richness estimation bias exists. Prior to diversity analyses, we tested for an effect of this difference in sampling size using rarefaction in the vegan package^58^. We sampled an equal number of reads from all sites (rarefied richness) and compared the results with observed patterns (observed richness). To account for PCR amplification bias and reaction stochasticity which affects the linear relationship between sequence abundance and sample abundance in highly diverse organisms, we used site presence-absence data for diversity (alpha and beta), and community composition analyses^59^. MOTU compositional differences among land-use types were examined using non-metric multidimensional scaling (nMDS) and the Jaccard index coefficient in the vegan package^58^. Alpha (α)-diversity was estimated as the number of observed MOTUs per site. Beta (β)-diversity was computed as multi-site Sorensen and Simpson indices using the betapart 1.3.package^60^. Beta-diversity calculations between land-use pairs were computed using 15 random sites from the total number of sites for each land-use type, and resample them 1000 times. We then decomposed the among-sites β-diversity into its turnover (species replacement from site to site) and nestedness (species gain/loss from sites) components. We evaluated differences in rate of species turnover between sites for plantations and for forests by regressing turnover against pairwise geographic distance (Mantel test^58^) and testing for a difference in slopes. Pairwise and three-way differences in MOTU diversity among land-use types were evaluated using the nonparametric multiple comparison function (*dunn.test*) implemented in the R package dunn.test 1.2.4^61^. The dunn.test is equivalent to the Kruskall–Wallis and pair-wise Mann–Whitney post hoc tests with Bonferroni correction. These analyses were performed in R^62^.

## Additional Information

**How to cite this article**: Beng, K. C. *et al.* The utility of DNA metabarcoding for studying the response of arthropod diversity and composition to land-use change in the tropics. *Sci. Rep.* **6**, 24965; doi: 10.1038/srep24965 (2016).

## Supplementary Material

Supporting Information

## Figures and Tables

**Table 1:** Characteristics of six mock communities (library 1–6) after *in silico* sequencing simulation and OTU picking.

Mock community	1	2	3	4	5	6
No. of reads	200,000	200,000	400,000	400,000	600,000	600,000
No. of reference genomes	25	40	25	40	25	40
No. of merged reads	2232	1755	4645	3494	6974	5117
Reads passed filtering (%)	66.0	61.8	63.8	62.0	64.2	62.3
No. of unique sequences	603	517	948	879	1271	1169
No. of singletons	458	396	666	646	906	861
OTUs_97% similarity	25	36	25	37	25	37

**Table 2:** Ordination (nMDS) results illustrating the effects of environmental gradients on MOTU composition across four land-use types.

	NMDS1	NMDS2	r2	Pr(>r)	
Distance between pairs	0.96	−0.26	0.23	0.000	***
Elevation	−0.97	0.23	0.86	0.000	***
Slope	−0.33	−0.94	0.14	0.006	**
Litter thickness	−0.51	−0.85	0.53	0.000	***
Topography	−0.47	−0.88	0.04	0.219	
Canopy	−0.27	0.96	0.59	0.000	***
Total Carbon	−0.58	−0.81	0.11	0.026	*
Total Nitrogen	0.34	0.93	0.09	0.056	.
Total Phosphorus	−0.02	0.99	0.20	0.001	**
Total Potassium	0.41	0.90	0.03	0.359	
Tannin content	−0.63	−0.76	0.07	0.101	
Coarse fiber content	−0.02	−0.99	0.35	0.000	***
Lignin content	−0.52	−0.85	0.10	0.023	*

Significant codes: 0 ‘***’ 0.001 ‘**’ 0.01 ‘*’ 0.05 ‘.’ 0.1 ‘ ’ 1. Number of permutations: 1000.

**Table 3:** Alpha diversity differences among the three main land-use types (forest, rubber and tea) for all MOTUs combined and for individual arthropod groups.

	X^2^	P	Pairwise comparisons	Z	P
All MOTUs	6.93	0.03	Forest vs Rubber	2.61	0.01
			Forest vs Tea	0.52	ns
			Rubber vs Tea	−1.83	ns
Arachnida	11.48	0.0001	Forest vs Rubber	1.92	ns
			Forest vs Tea	3.27	0.001
			Rubber vs Tea	1.12	ns
Blattodea	0.32	ns	Forest vs Rubber	0.05	ns
			Forest vs Tea	0.55	ns
			Rubber vs Tea	0.42	ns
Coleoptera	21.91	0.0001	Forest vs Rubber	4.60	0.0001
			Forest vs Tea	2.33	0.02
			Rubber vs Tea	−2.01	0.06
Diptera	14.55	0.0001	Forest vs Rubber	3.08	0.0031
			Forest vs Tea	3.14	0.0025
			Rubber vs Tea	0.00	ns
Hemiptera	22.07	0.0001	Forest vs Rubber	4.20	0.0001
			Forest vs Tea	−0.58	ns
			Rubber vs Tea	−4.17	0.0001
Hymenoptera	3.11	ns	Forest vs Rubber	−0.81	ns
			Forest vs Tea	1.20	ns
			Rubber vs Tea	1.74	ns
Chilopoda	1.44	ns	Forest vs Rubber	−0.67	ns
			Forest vs Tea	−1.16	ns
			Rubber vs Tea	−0.40	ns
Orthoptera	21.79	0.0001	Forest vs Rubber	−1.30	ns
			Forest vs Tea	−4.66	0.0001
			Rubber vs Tea	−2.85	0.01

Differences are based on Kruskal-Wallis rank sum test and Mann-Whitney U test with Bonferroni correction.

**Table 4:** Regression coefficients (Mantel statistic) for all arthropods MOTUs and for each arthropod group, with their significance level, for species turnover in forests and plantations (rubber and tea) plotted against geographic distances between sampling locations.

	All MOTUs	Blattodea	Coleoptera	Diptera	Hemiptera	Hymenoptera	Orthoptera	Arachnida	Chilopoda
Rubber	0.03	0.07	0.13	0.09	0.08	−0.14	−0.03	−0.11	0.04
**P value**	**ns**	**ns**	**Ns**	**ns**	**ns**	**ns**	**ns**	**ns**	**ns**
Rubber-forest	0.09	0.17	0.27	0.02	0.18	−0.11	−0.15	−0.17	0.05
**P value**	**ns**	**ns**	**<0.01**	**ns**	**ns**	**ns**	**ns**	**ns**	**ns**
Tea	0.24	0.05	0.12	0.19	0.17	0.17	0.22	0.28	0.01
**P value**	**<0.001**	**ns**	**ns**	**<0.01**	**<0.05**	**<0.05**	**<0.01**	**<0.001**	**ns**
Tea-forest	0.34	0.14	0.36	0.18	0.21	0.21	−0.01	0.2	0.10
**P value**	**<0.001**	**ns**	**<0.001**	**<0.01**	**<0.01**	**<0.01**	**ns**	**<0.01**	**ns**
