# Supplementary material for: The utility of DNA metabarcoding for studying the response of arthropod diversity and composition to land-use change in the tropics
Source: Sci Rep. 2016 Apr 26;6:24965. doi: 10.1038/srep24965 (PMC4844954; doi:10.1038/srep24965)
Supplement: Supporting Information [file srep24965-s1.pdf]

SUPPORTING INFORMATION

The utility of DNA metabarcoding for studying the response of arthropod diversity and composition to land-use change in the tropics

Kingsly Chuo Beng[1,2*], Kyle W. Tomlinson[1], Xian Hui Shen[1,2], Yann Surget-Groba[3,4], Alice C. Hughes[1], Richard T. Corlett[1] and J.W. Ferry Slik[5]

[1]Center for Integrative Conservation, Xishuangbanna Tropical Botanical Garden, Chinese Academy of Sciences, Menglun, Mengla, Yunnan, 666303, China.

[2]University of Chinese Academy of Sciences, No.19A Yuquan Road, Beijing 100049, China

[3]Institut des Sciences de la Forêt Tempérée, Université du Québec en Outaouais, 58, Rue Principale, Ripon, Québec J0V 1V0, Canada.

[4]Quebec Centre for Biodiversity Science, McGill University, 1205 Dr. Penfield Avenue, Montreal, Quebec, H3A 1B1, Canada

[5]Faculty of Science, Universiti Brunei Darussalam, Jln. Tungku Link, Gadong, BE1410, Brunei Darussalam.

*Corresponding author: (email: bengkingsly2000@yahoo.com; Tel: +86-13628810320)

A

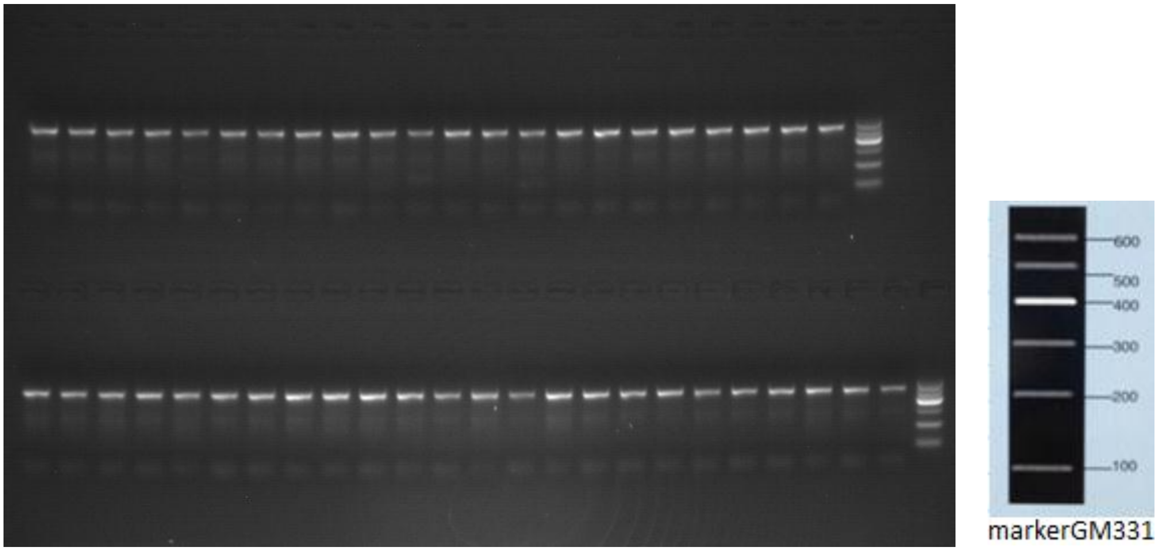

B

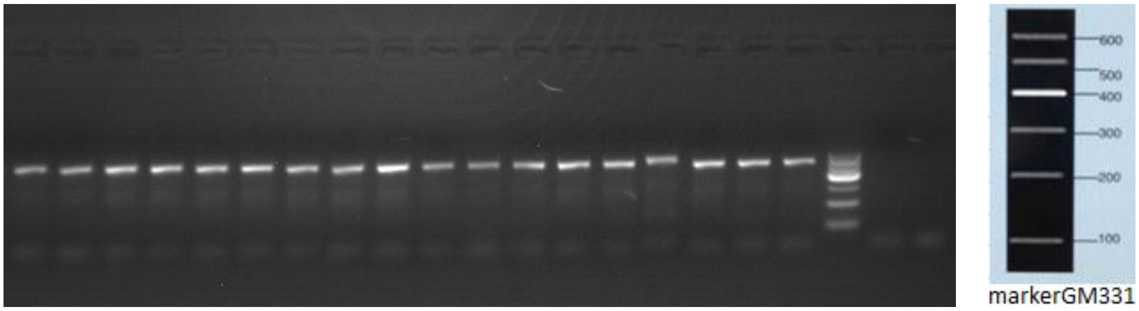

Fig S1 Gel electrophoresis for (A): Bulk arthropod samples and (B): Individual arthropod samples (from right to left; Hymenoptera sp1 & sp2, Araneae sp1 & sp2, Blattodea sp1 & sp2, Mantodea sp1 & sp2, Coleoptera sp1 & sp2, Chilopoda sp1 and sp2, Orthoptera sp1 & sp2, Hemiptera sp1 & sp2, Lepidoptera sp1 & sp2.

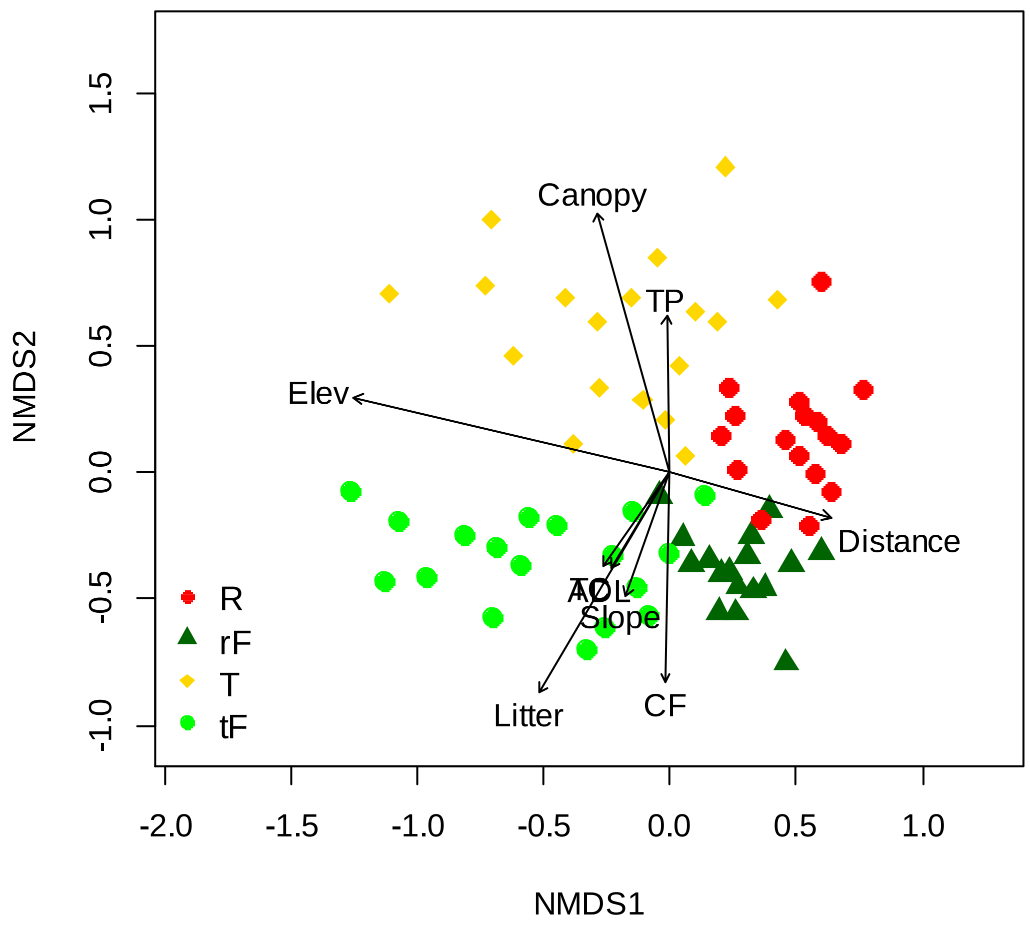

Figure S2 Ordination (nMDS) plot illustrating the similarities and differences in MOTU composition across four land-use types and the effects of environmental variables. The variables were fitted to the ordination plot using 1000 permutations. Elev = elevation, Canopy = canopy cover, TP = total Phosphorus, TC = total Carbon, ADL = lignin, Litter = leaf litter thickness, CF = coarse fiber, Distance= horizontal distance between forest-plantation pairs

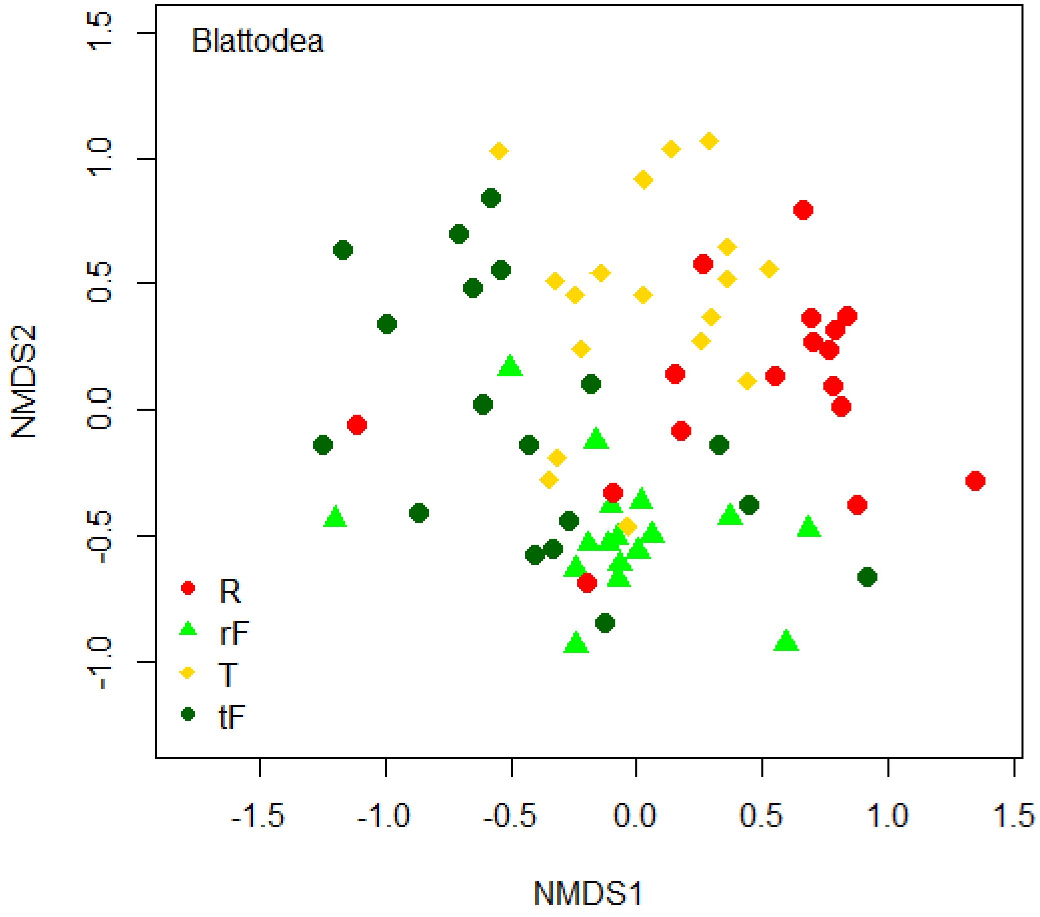

Figure S3 Ordination (nMDS) plots illustrating the similarities and differences in MOTU composition from the two matched forest-plantation pairs

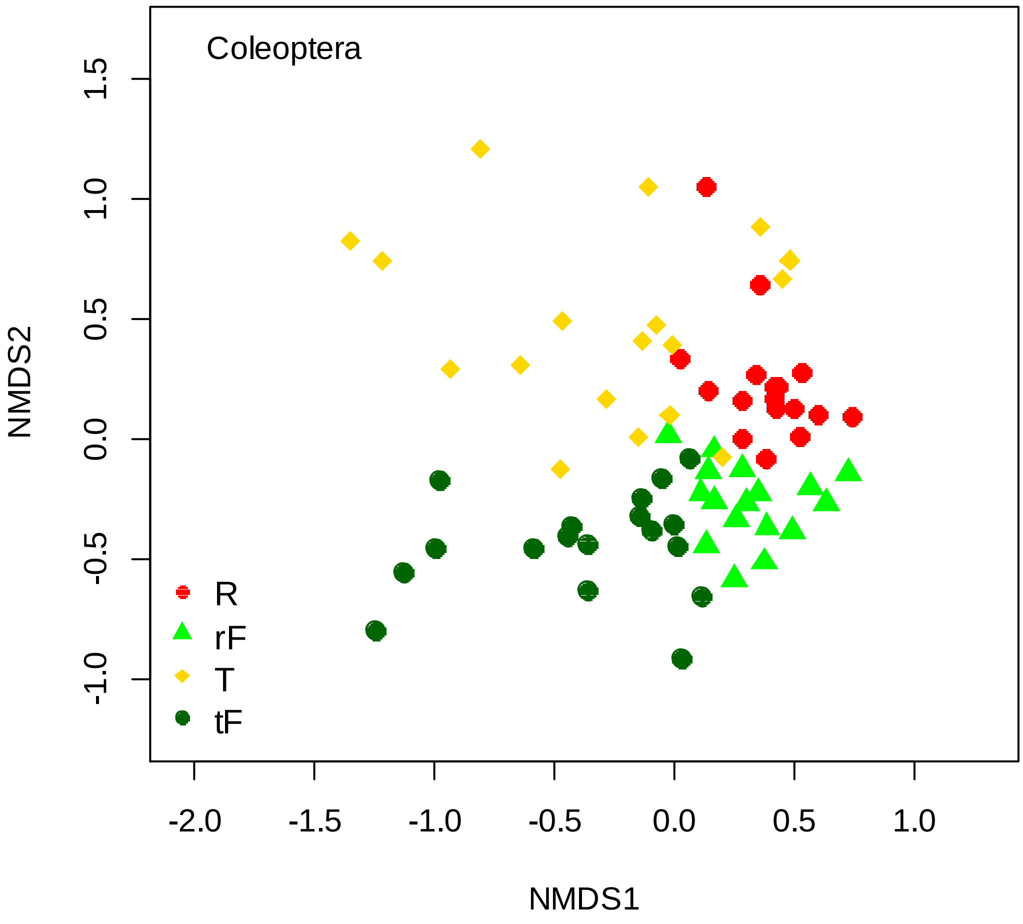

Figure S4 Ordination (nMDS) plots illustrating the similarities and differences in MOTU composition from the two matched forest-plantation pairs

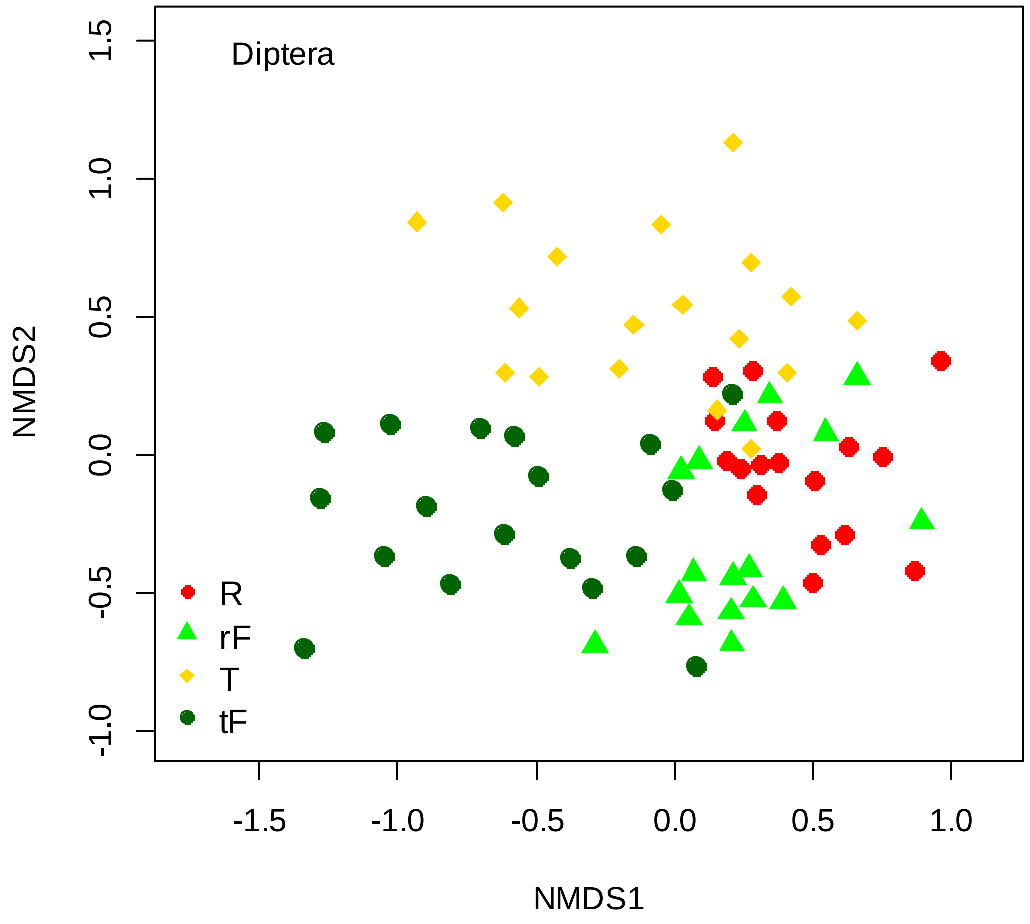

Figure S5 Ordination (nMDS) plots illustrating the similarities and differences in MOTU composition from the two matched forest-plantation pairs

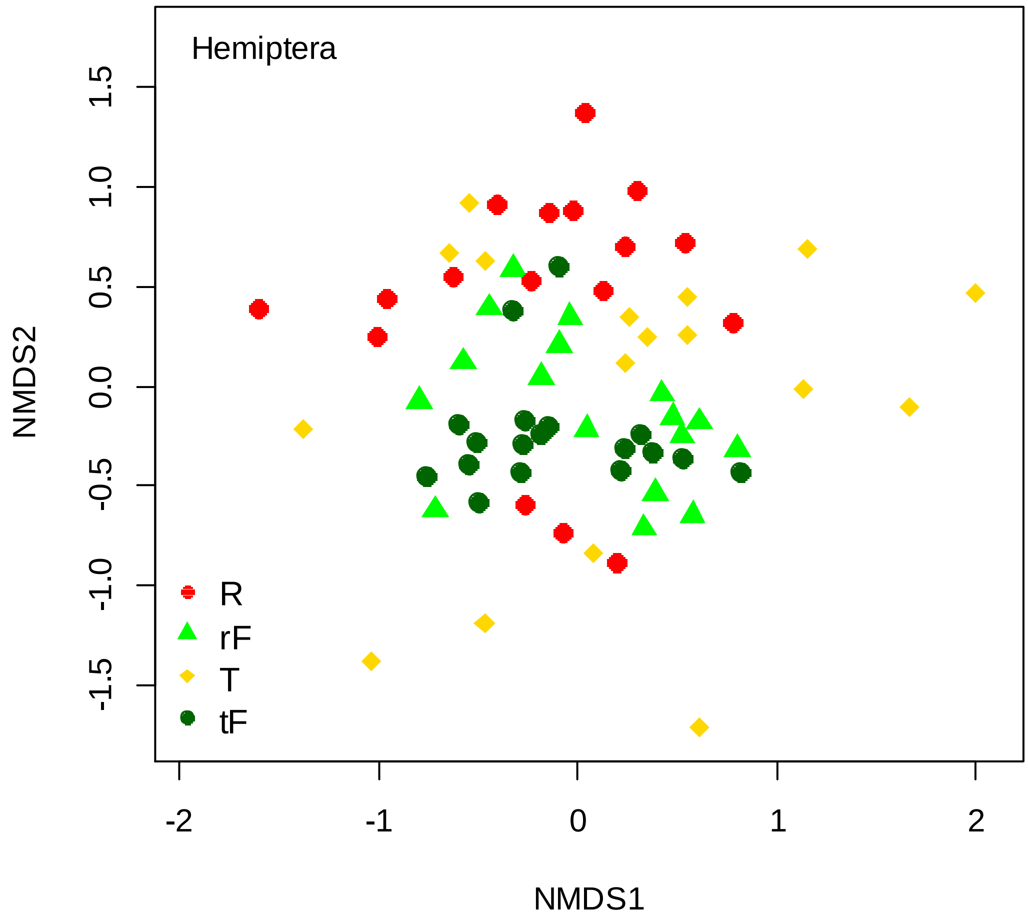

Figure S6 Ordination (nMDS) plots illustrating the similarities and differences in MOTU composition from the two matched forest-plantation pairs

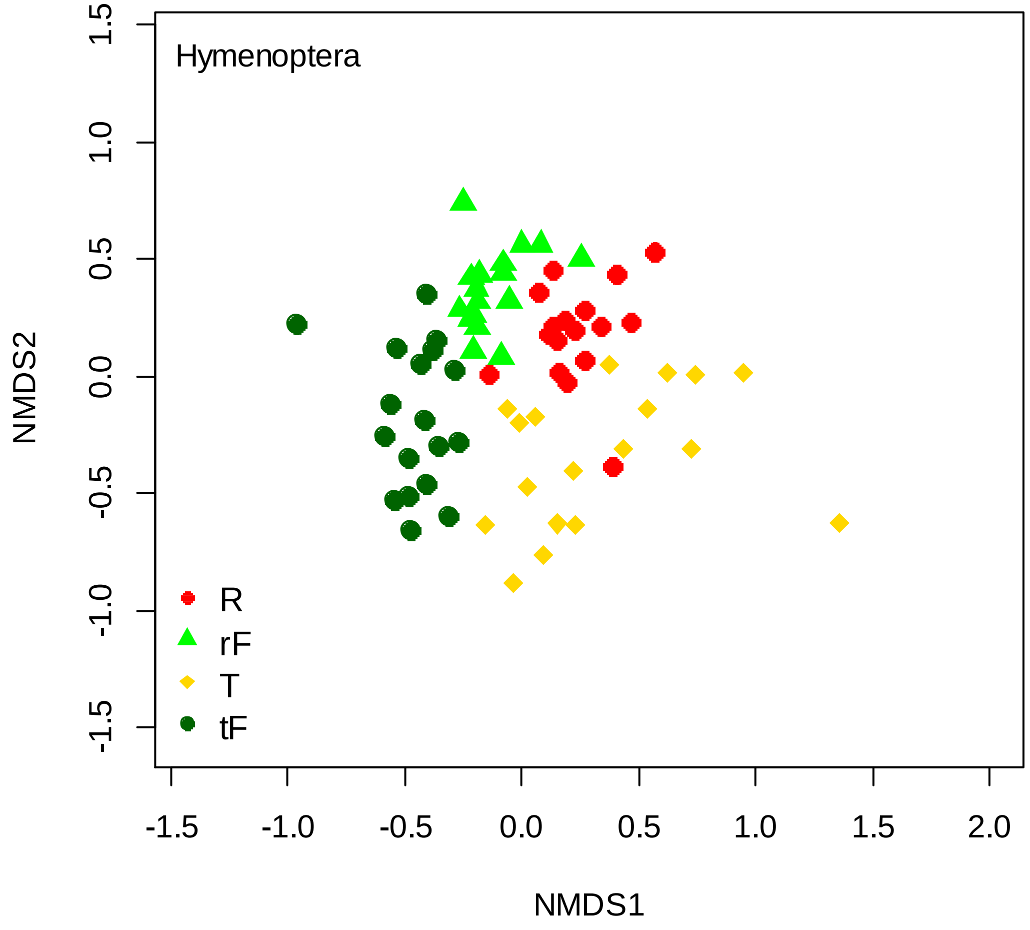

Figure S7 Ordination (nMDS) plots illustrating the similarities and differences in MOTU composition from the two matched forest-plantation pairs

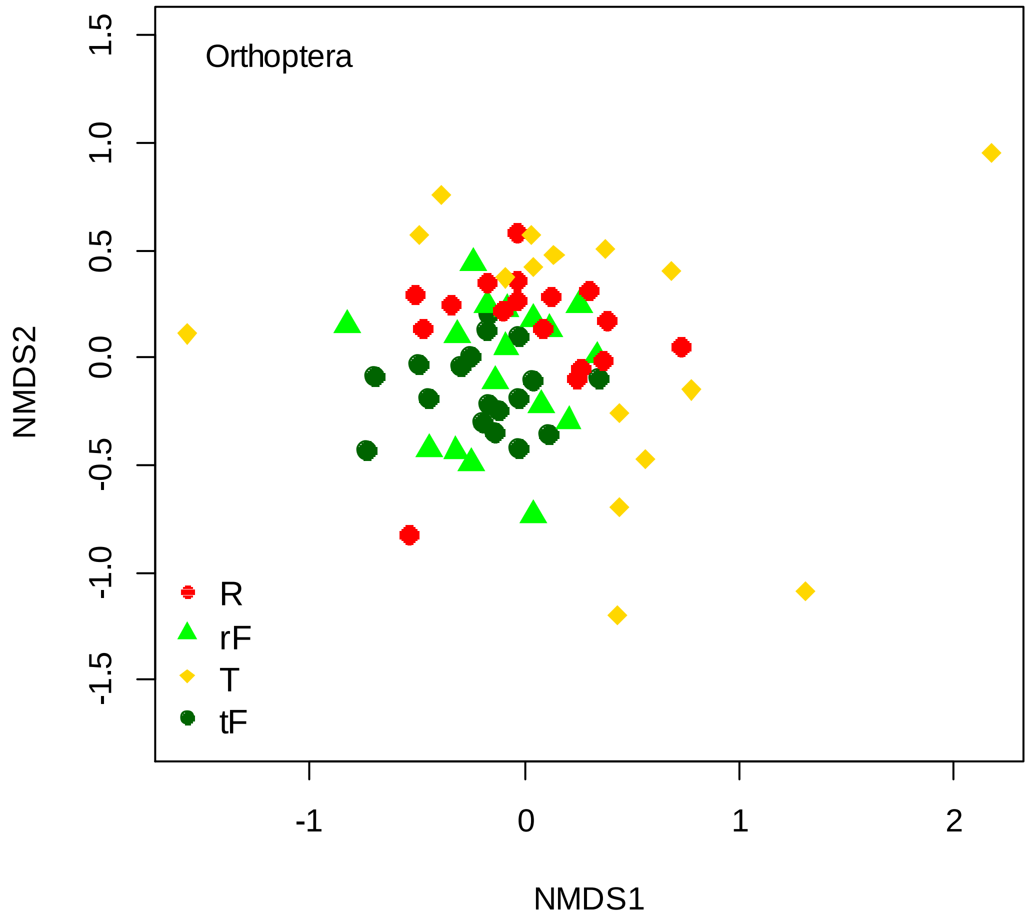

Figure S8 Ordination (nMDS) plots illustrating the similarities and differences in MOTU composition from the two matched forest-plantation pairs

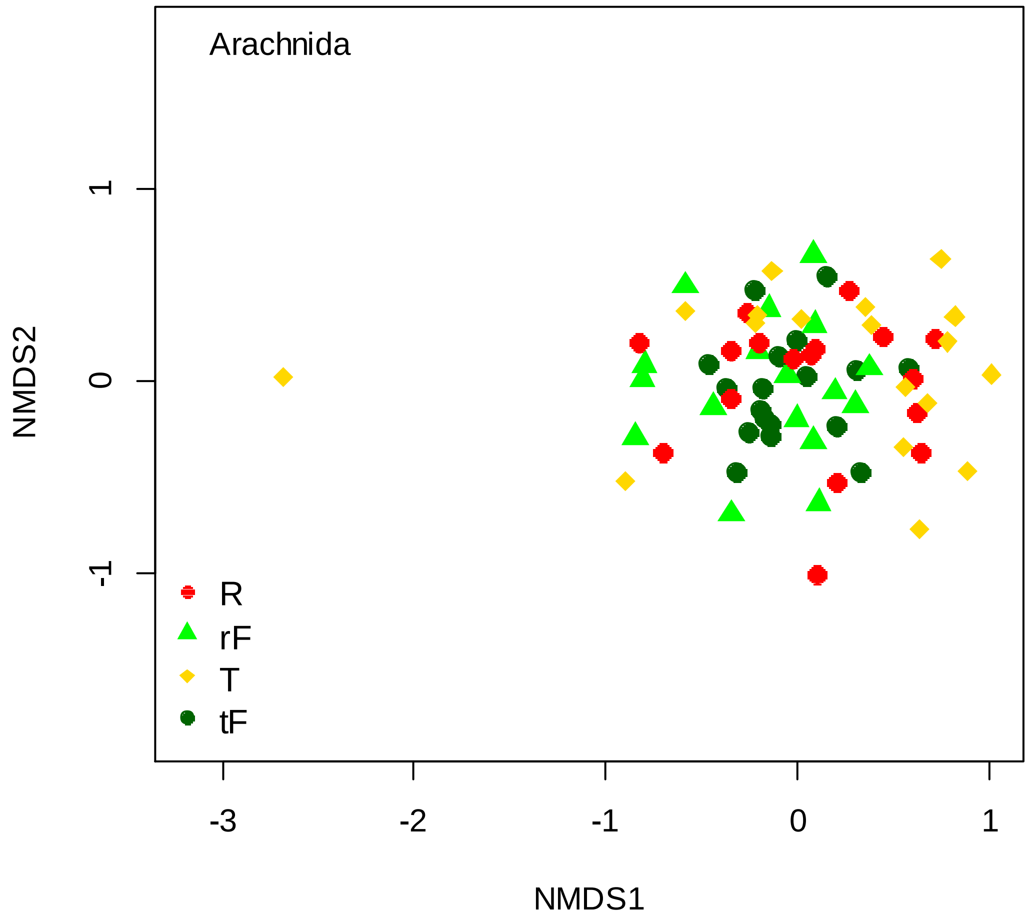

Figure S9 Ordination (nMDS) plots illustrating the similarities and differences in MOTU composition from the two matched forest-plantation pairs

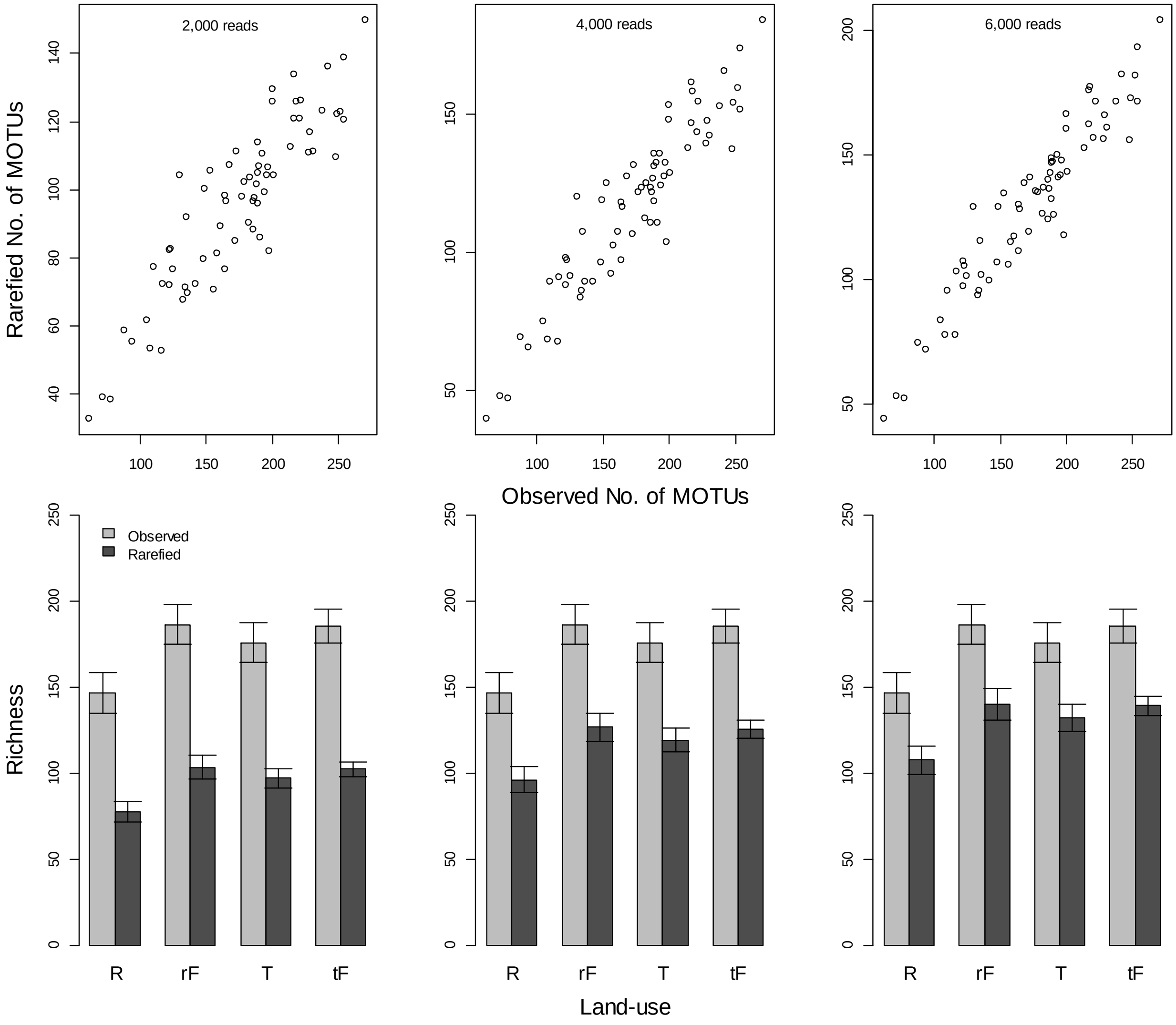

Figure S10 Relationship between observed and rarefied MOTU richness. Rarefaction was performed by randomly drawing 2,000; 4,000; and 6,000 reads (upper graphs) from each of the 70 sampled sites.

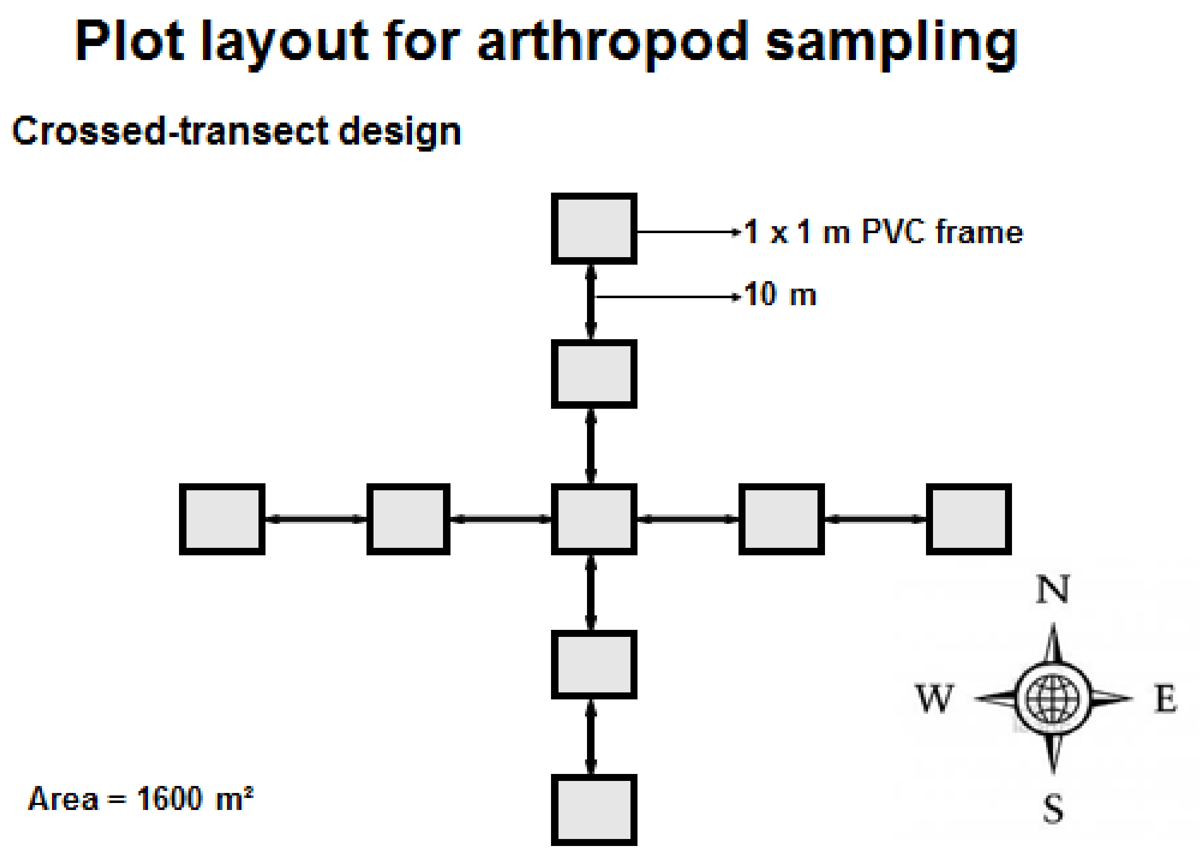

Figure S11 Arthropod sampling plot design. Drawing was made using Microsoft PowerPoint 2010.

Table S1 Community composition of the 37 reference sequences used to simulate six mock communities

Order	Family	Species
Hemiptera	Achilidae	*Magadha taibaishanensis*
Lepidoptera	Sphingidae	*Hyles_annei*
Decapoda	Lithodidae	*Paralithodes_camtschaticus*
Orthoptera	Gryllidae	*Gryllus sp.*
Lepidoptera	Lycaenidae	*Lachnocnema sp.*
Diptera	Culicinae	*Borichinda cavernicola*
Lepidoptera	Castniidae	*Telchin licus*
Arachnida	Ixodidae	*Dermacentor reticulatus*
Lepidoptera	Lycaenidae	*Wagimo signata*
Diptera	Culicinae	*Culiseta longiareolata*
Lepidoptera	Nymphalidae	*Melitaea didyma*
Pantopoda	Colossendeidae	*Colossendeis macerrima*
Lepidoptera	Nymphalidae	*Mycalesis janardana*
Diptera	Agromyzidae	*Ophiomyia nasuta*
Diptera	Simuliidae	*Simulium balcanicum*
Diptera	Psychodidae	*Lutzomyia (Helcocyrtomyia) sp.*
Diptera	Drosophilidae	*Leucophenga angusta*
Lepidoptera	Nymphalidae	*Eunica viola*
Lepidoptera	Crytophasidae	*Opisina arenosella*
Lepidoptera	Nymphalidae	*Nessaea obrinus*
Hemiptera	Anthocoridae	*Anthocoris expansus*
Ixodida	Ixodidae	*Rhipicephalus pumilio*
Hemiptera	Cercopidae	*Locris rubra*
Ephemeroptera	Heptageniidae	*Epeorus vitreus*

Coleoptera	Lampyridae	*Pyractomena borealis*
Orthoptera	Acrididae	*Prumna mandshurica*
Mantodea	Mantidae	*Elmantis nira*
Lepidoptera	Nymphalidae	*Eresia Letitia*
Orthoptera	Acrididae	*Aerochoreutes carlinianus*
Hemiptera	Lygaeidae	*Spilostethus pandurus*
Diptera	Drosophilidae	*Drosophila melanogaster*
Diptera	Culicidae	*Uranotaenia macfarlanei*
Hymenoptera	Mymaridae	*Anagrus ustulatus*
Ixodida	Ixodidae	*Rhipicephalus haemaphysaloides*
Coleoptera	Carabidae	*Trechus uhagoni*
Diptera	Culicidae	*Borichinda cavernicola*
Hymenoptera	Vespidae	*Polybia sericea*

Table S2 Validated mock community sequences showing the category (Classification), percent identity between the query sequence and the top hit in the reference database (Top hit), percent identity between the query sequence and the maximum parsimony model (MPM) and matched species in reference database. **Perfect**: 100% match to a reference sequence. Size gives the number of reads assigned to each OTU. Results are for one mock community (6).

OTU	Classification	Top hit	MPM	Matched species(s) in reference database
OTU_9;size=59	perfect	100	100	GBCL2738-07|Pyractomena
OTU_8;size=59	perfect	100	100	MDA578-08|Epeorus
OTU_2;size=178	perfect	100	100	GBCMD13542-13|Paralithodes
OTU_5;size=79	perfect	100	100	JQ737085.1;Rhipicephalus_haemaphysaloides;
OTU_7;size=60	perfect	100	100	GBMIN17748-13|Leucophenga
OTU_6;size=90	perfect	100	100	GBMOR766-13|Gryllus|COI-5P|JX436481
OTU_10;size=62	perfect	100	100	GBMH5085-08|Aerochoreutes
OTU_11;size=59	perfect	100	100	GBMH8038-10|Elmantis
OTU_4;size=176	perfect	100	100	AJ749430.1;Hyles_annei;
OTU_12;size=49	perfect	100	100	GBMIN22936-13|Anthocoris
OTU_3;size=141	perfect	100	100	EF370413.1;Borichinda_cavernicola;
OTU_15;size=50	perfect	100	100	KF894745.1;Magadha_taibaishanensis;
OTU_14;size=57	perfect	100	100	ANGEN047-14|Tropidothorax|COI-5P
OTU_1;size=234	perfect	100	100	HM193878.1;Rhipicephalus_pumilio;
OTU_13;size=78	perfect	100	100	KC921257.1;Lutzomyia_(Helcocyrtomyia);
OTU_16;size=60	perfect	100	100	HM193885.1;Dermacentor_reticulatus;
OTU_17;size=46	perfect	100	100	GBDP3916-07|Ophiomyia
OTU_18;size=70	perfect	100	100	GBMIN14844-13|Telchin
OTU_20;size=73	perfect	100	100	FJ531676.1;Prumna_mandshurica;
OTU_23;size=54	perfect	100	100	GBLN1858-09|Melitaea
OTU_25;size=43	perfect	100	100	GBCL14999-13|Trechus
OTU_22;size=35	perfect	100	100	GBDPD294-14|Drosophila
OTU_19;size=42	perfect	100	100	JQ728133.1;Uranotaenia_macfarlanei;
OTU_26;size=46	perfect	100	100	GBGL19546-15|Lachnocnema
OTU_24;size=44	perfect	100	100	GBDCU607-12|Culiseta
OTU_21;size=39	perfect	100	100	GBA20055-14|Colossendeis
OTU_27;size=37	perfect	100	100	GBMIN10292-12|Locris
OTU_28;size=51	perfect	100	100	GBLN0423-06|Eresia
OTU_34;size=14	perfect	100	100	GBMIN10748-12|Anagrus
OTU_29;size=33	perfect	100	100	GBMIN37947-13|Wagimo
OTU_30;size=55	perfect	100	100	JN900474.1;Opisina_arenosella;
OTU_31;size=30	perfect	100	100	GBMIN20229-13|Simulium
OTU_32;size=34	perfect	100	100	GBLN2848-10|Eunica
OTU_35;size=16	perfect	100	100	GBMIN37842-13|Mycalesis
OTU_37;size=22	perfect	100	100	KC921295.1;Lutzomyia_(Helcocyrtomyia);
OTU_36;size=37	perfect	100	100	GBLN2818-10|Nessaea

OTU_33;size=12	perfect	100	100	GBAH1587-06|Polybia

Table S3 The number of quality filtered reads and their mean fragment length across 70 sample sites.

Habitat	Landuse	Latitude	Longitude	Barcode	No. filtered reads	Mean read length
tF01	Forest	22.385212	100.958524	GCATCGT	47,726	398.3
tF02	Forest	22.209488	101.315989	CACTCTA	15,342	397.7
tF03	Forest	22.085498	101.192795	CTGAGAC	14,965	396
tF04	Forest	22.102885	101.342195	TCATCTG	46,799	395.5
tF05	Forest	22.151386	101.45907	GCGTGTG	48,335	395.6
tF06	Forest	21.992465	101.10544	GTGCGAT	41,972	397.5
tF07	Forest	22.239103	100.394079	GCTAGCA	48,835	398.9
tF08	Forest	22.098249	100.547548	TACTAGT	51,139	398.4
tF09	Forest	21.901733	100.541062	ATATGTA	32,773	399.9
tF10	Forest	21.834458	100.539369	ATGTGTG	33,745	398.1
tF11	Forest	21.584436	100.426162	CATGTCG	41,985	399.2
rF12	Forest	21.223269	101.690125	TCGTATG	45,117	388.6
tF13	Forest	21.904397	100.146904	AGCTCAT	41,167	398.6
tF14	Forest	21.945943	100.129664	AGAGACA	51,631	399.1
tF15	Forest	22.224714	101.34951	AGTCGCG	24,685	398.6
rF16	Forest	21.697214	101.278743	GACTGTC	21,958	398
rF17	Forest	21.97157	101.274464	GAGCATA	49,319	348
rF18	Forest	21.883692	101.295208	TGTGTCA	49,097	391.1
rF19	Forest	21.499948	101.53474	GCATCGT	50,964	398.5
rF20	Forest	22.158267	100.783992	ATATGTA	37,225	396.7
rF21	Forest	22.159294	100.945697	GCGTCGC	39,001	397.1
rF22	Forest	22.015584	100.97392	AGTGCTG	36,063	357.6
rF23	Forest	21.912049	101.134264	ACTCGCT	24,744	399.5
rF24	Forest	21.846596	101.379262	GCGTGTG	46,400	396.9
rF25	Forest	21.909728	100.740862	TACTAGT	30,862	399.6
rF26	Forest	22.154768	100.672528	ATGTGTG	21,099	397.6
rF27	Forest	21.674871	100.722957	GTCTGAC	44,819	398.7
tF28	Forest	21.970419	101.479784	GCTAGCA	34,618	397.3
tF29	Forest	22.029231	100.618368	TCATCTG	38,203	396.6
tF30	Forest	22.525476	101.103012	GTGCGAT	33,628	398.7
rF31	Forest	21.953084	101.257076	GATATGA	42,602	397.8
rF32	Forest	21.919592	101.239585	TGTGTAT	40,802	399
rF33	Forest	21.948294	101.298306	CACTCTA	35,837	396.3
rF34	Forest	21.916449	101.21363	CTGAGAC	37,124	390.1
rF35	Forest	21.917029	101.271519	ATCTAGT	38,267	398.2
R12	Rubber	21.225437	101.693636	ATAGTGA	25,719	391
R16	Rubber	21.698906	101.278556	CACAGCA	37,585	396.1

R17	Rubber	21.97108	101.270274	TGCGTAG	39,051	364.1
R18	Rubber	21.884041	101.294311	GTACATA	30,432	360.6
R19	Rubber	21.499337	101.535627	GTGCTCG	17,808	397.3
R20	Rubber	22.158707	100.785414	GTGTCTG	23,766	390.8
R21	Rubber	22.160814	100.946758	TCACATC	34,553	398.6
R23	Rubber	21.916058	101.134858	CTCAGAT	36,687	396.2
R24	Rubber	21.84609	101.380471	GATATCG	22,918	396.7
R25	Rubber	21.910717	100.740436	GTATAGT	27,902	395.1
R26	Rubber	22.154524	100.669341	ACATATA	19,278	399.8
R27	Rubber	21.67275	100.723944	AGAGACA	50,181	400
R31	Rubber	21.953313	101.253649	TGTGTAT	28,856	398.6
R32	Rubber	21.920514	101.240265	TGTGTCA	44,193	382.8
R33	Rubber	21.945426	101.302209	GACTGTC	40,151	396.9
R34	Rubber	21.920158	101.214222	GATATCG	31,662	396.2
R35	Rubber	21.910556	101.272179	GTGTCTG	34,105	358.1
T01	Tea	22.386482	100.957753	CATGTCG	24,745	369.5
T02	Tea	22.212192	101.313751	AGTGCTG	29,955	388.8
T03	Tea	22.087136	101.194285	AGTCGCG	40,065	392
T04	Tea	22.103814	101.341934	ATCTAGT	23,435	393.9
T05	Tea	22.152392	101.460156	TCACATC	52,723	395.6
T06	Tea	21.992713	101.103068	AGCTCAT	30,112	397.8
T07	Tea	22.239333	100.392942	ATAGTGA	49,322	395.1
T08	Tea	22.098209	100.548005	CTCAGAT	51,937	397.9
T09	Tea	21.900615	100.541164	TGCGTAG	24,821	398.8
T10	Tea	21.835022	100.53975	TCGTATG	25,383	398.4
T11	Tea	21.586601	100.425055	GTATAGT	51,649	397.8
T13	Tea	21.904553	100.147851	GAGCATA	46,277	363.1
T14	Tea	21.944589	100.12863	ACATATA	54,438	396.4
T15	Tea	22.223876	101.3495	GTACATA	44,649	388.8
T22	Tea	22.014433	100.973694	GCGTCGC	43,431	330
T28	Tea	21.969301	101.478696	ACTCGCT	39,951	359.5
T29	Tea	22.027712	100.618467	GTGCTCG	29,883	396.7
T30	Tea	22.524253	101.102906	CACAGCA	27,008	373.7

Table S4 Comparison between two taxonomic assignment algorithms (UTAX and USEARCH) for MOTUs assigned to arthropod order Hymenoptera. Out of the 261 MOTUs assigned by UTAX, 253 were also assigned by USEARCH (97% correspondence). Taxonomic assignment for eight OTUs (marked in bold and asterisks) was different for both algorithms.

	UTAX	USEARCH	
OTU_ID	Order (Confidence value)	Reference database top hit	Identity
OTU_1001;size=19;	Hymenoptera(99.5)	GMSUE180-14|Hymenoptera|COI-5P	98.3
OTU_1008;size=19;	Hymenoptera(53.9)	BBFOA107-10|Formicidae|COI-5P|HQ928626	85.1
OTU_1017;size=41;	Hymenoptera(99.6)	ASANQ180-09|Formicidae|COI-5P|GU710912	99.5
OTU_1019;size=28;	Hymenoptera(57.9)	GMMGN787-14|Hymenoptera|COI-5P	97.1
OTU_1033;size=29;	Hymenoptera(92.1)	ASANZ486-10|Formicidae|COI-5P|HQ547736	90.1
OTU_1046;size=35;	Hymenoptera(40.8)	ASANR616-09|Monomorium	82
OTU_1067;size=19;	Hymenoptera(65.7)	CNGRL255-13|Encyrtidae|COI-5P|KR789888	87.9
OTU_1074;size=24;	Hymenoptera(75.1)	ASANA614-06|Hymenoptera|COI-5P	87.8
OTU_109;size=1263;	Hymenoptera(43.2)	GMBCG389-15|Hymenoptera|COI-5P	97.9
OTU_1090;size=16;	Hymenoptera(61.8)	ASPNA1724-11|Formicidae|COI-5P	91.6
OTU_1095;size=16;	Hymenoptera(63.7)	MAHYM766-12|Hymenoptera|COI-5P	89.3
OTU_1098;size=18;	Hymenoptera(33.3)	ASANA553-06|Hymenoptera|COI-5P	81.9
OTU_1109;size=18;	Hymenoptera(40.8)	ASPNA1409-10|Formicidae|COI-5P|HQ551086	86.6
OTU_1117;size=24;	Hymenoptera(99.5)	GBMIN19810-13|Prenolepis	98.3
OTU_1118;size=36;	Hymenoptera(33.3)	ASBEZ463-11|Formicidae|COI-5P	79.2
OTU_1121;size=26;	Hymenoptera(99.8)	WOMIC091-10|Neoclarkinella|COI-5P|JN282227	99.8
OTU_1126;size=19;	Hymenoptera(94.8)	GMMGC015-14|Hymenoptera|COI-5P	95.5
OTU_1134;size=17;	Hymenoptera(43.2)	ASAZT049-11|Formicidae|COI-5P	84.1
OTU_1149;size=17;	Hymenoptera(49.8)	ACGAG525-11|Pyramica	82.9
OTU_1151;size=15;	Hymenoptera(70.2)	BBHYA3292-12|Hymenoptera|COI-5P	86.4
OTU_1163;size=57;	Hymenoptera(80.6)	ASPAL025-09|Hymenoptera|COI-5P	90.8
OTU_1199;size=16;	Hymenoptera(70.2)	GBMIN30395-13|Empria	77.9
OTU_1223;size=29;	Hymenoptera(53.9)	ASANP367-09|Formicidae|COI-5P|GU710267	82.6
OTU_1255;size=21;	Hymenoptera(63.7)	ASANA239-06|Hymenoptera|COI-5P|KF605021	83.9
OTU_1283;size=12;	Hymenoptera(61.8)	GBMIN25819-13|Hymenoptera|COI-5P|JQ344538	98.1
OTU_1288;size=12;	Hymenoptera(98.0)	CNGBO738-14|Baryscapus|COI-5P|KR405478	92.5
OTU_1307;size=33;	Hymenoptera(98.6)	MGABB398-10|Formicidae|COI-5P|HM893002	92.3
OTU_1326;size=12;	Hymenoptera(99.3)	GBAHF101-13|Polyrhachis	94.8
OTU_1331;size=14;	Hymenoptera(60.0)	ASAND755-10|Formicidae|COI-5P|JN283071	85.1
OTU_1360;size=15;	Hymenoptera(23.9)	ASBEZ373-10|Formicidae|COI-5P|HQ548139	80.9
OTU_138;size=1452;	Hymenoptera(67.8)	GBMIN25144-13|Hymenoptera|COI-5P|JQ345027	91.6
**OTU_1393;size=15;***	**Hymenoptera(40.8)**	**WMGSI354-10|Trombidiformes|COI-5P|HQ584522**	**77.4**
OTU_1395;size=10;	Hymenoptera(77.9)	ASPNA2359-11|Hymenoptera|COI-5P	88.8
OTU_143;size=1082;	Hymenoptera(33.3)	GMBCC1926-15|Hymenoptera|COI-5P	91.1

OTU_145;size=1819;	Hymenoptera(43.2)	ASPNA1211-10|Formicidae|COI-5P|HQ550921	87.8
OTU_1467;size=14;	Hymenoptera(99.4)	GBAHF076-13|Myrmica	96.5
OTU_1497;size=9;	Hymenoptera(43.2)	ASMAS039-08|Camponotus	84.9
OTU_1501;size=11;	Hymenoptera(77.9)	ASAM3185-05|Hymenoptera|COI-5P	86.6
OTU_1540;size=13;	Hymenoptera(61.8)	ASPNA2314-11|Hymenoptera|COI-5P	89.3
OTU_1541;size=11;	Hymenoptera(30.9)	GMMGA466-14|Hymenoptera|COI-5P	85.8
OTU_1543;size=9;	Hymenoptera(28.8)	ASLAM2054-12|Crematogaster	81.5
OTU_1557;size=14;	Hymenoptera(49.8)	ASPNA2217-11|Hymenoptera|COI-5P	86.4
OTU_1561;size=8;	Hymenoptera(99.0)	GMMGH414-14|Hymenoptera|COI-5P	91.9
OTU_1563;size=8;	Hymenoptera(88.1)	GBAH11649-15|Nylanderia	93
OTU_1580;size=11;	Hymenoptera(61.8)	ASPNA1463-10|Formicidae|COI-5P|HQ551124	88.1
OTU_1582;size=8;	Hymenoptera(49.8)	ASPNA1724-11|Formicidae|COI-5P	92.3
OTU_1600;size=10;	Hymenoptera(67.8)	GBAH11701-15|Nylanderia	100
OTU_1601;size=10;	Hymenoptera(83.2)	MAHYM390-10|Hymenoptera|COI-5P|JF866256	86.8
OTU_1610;size=8;	Hymenoptera(99.9)	GBAHF097-13|Polyrhachis	100
OTU_1618;size=8;	Hymenoptera(49.8)	ASANY702-10|Formicidae|COI-5P|HQ547426	82.4
OTU_1629;size=8;	Hymenoptera(49.8)	ASANL126-10|Formicidae|COI-5P	86.4
OTU_164;size=1214;	Hymenoptera(61.8)	ASPAL025-09|Hymenoptera|COI-5P	89.1
OTU_1640;size=7;	Hymenoptera(40.8)	ASANI066-11|Formicidae|COI-5P|JN283519	80.6
OTU_1643;size=7;	Hymenoptera(96.6)	GBAH11704-15|Nylanderia	96.8
OTU_1650;size=7;	Hymenoptera(49.8)	ASPNA1201-10|Formicidae|COI-5P|HQ550911	82.1
OTU_1664;size=9;	Hymenoptera(35.8)	BCIFO1236-13|Hymenoptera|COI-5P	81.9
OTU_1667;size=14;	Hymenoptera(99.2)	GBAH2166-06|Odontoponera	92.3
OTU_1680;size=7;	Hymenoptera(56.0)	GBMIN25577-13|Hymenoptera|COI-5P|JQ345028	97.8
OTU_1698;size=11;	Hymenoptera(85.7)	GBAHF115-13|Pseudolasius	88.6
**OTU_1703;size=7;***	**Hymenoptera(60.0)**	**MAMOT462-10|Lepidoptera|COI-5P|HQ991154**	**78.4**
OTU_171;size=764;	Hymenoptera(88.1)	GBAH7030-10|Pachycondyla	90.8
OTU_1742;size=12;	Hymenoptera(72.5)	ASPAL025-09|Hymenoptera|COI-5P	90.3
OTU_1749;size=11;	Hymenoptera(99.7)	GBAHF331-13|Dolichoderus	100
OTU_1751;size=7;	Hymenoptera(61.8)	FSA627-12|Hymenoptera|COI-5P	92.1
OTU_1755;size=7;	Hymenoptera(72.5)	GMCRD114-13|Hymenoptera|COI-5P	89.3
OTU_1762;size=7;	Hymenoptera(80.6)	GBAHF113-13|Cataglyphis|COI-5P|JQ681054	100
OTU_1765;size=12;	Hymenoptera(99.4)	MAMTN340-13|Hymenoptera|COI-5P	88.8
OTU_1789;size=6;	Hymenoptera(51.9)	AUSBC287-12|Hymenoptera|COI-5P	83.4
OTU_1799;size=6;	Hymenoptera(47.7)	ASKBB059-14|Monomorium	86.6
OTU_1807;size=8;	Hymenoptera(49.8)	ASANJ1179-11|Hymenoptera|COI-5P	84.1
OTU_1808;size=8;	Hymenoptera(99.2)	GBAHF076-13|Myrmica	95.5
OTU_1816;size=8;	Hymenoptera(63.7)	GBMIN25144-13|Hymenoptera|COI-5P|JQ345027	91.9
OTU_1834;size=19;	Hymenoptera(98.4)	MAHYM468-10|Hymenoptera|COI-5P|JF866317	88.8
OTU_1839;size=6;	Hymenoptera(61.8)	NZHYM361-10|Formicidae|COI-5P|JN283828	93.8
OTU_1843;size=6;	Hymenoptera(65.7)	ASPNA1308-10|Formicidae|COI-5P|HQ551004	90.1
OTU_1849;size=8;	Hymenoptera(57.9)	GMBCD243-15|Hymenoptera|COI-5P	92.6
OTU_1873;size=6;	Hymenoptera(57.9)	GMMGD2127-14|Hymenoptera|COI-5P	92.2

OTU_189;size=588;	Hymenoptera(77.9)	ASANA565-06|Hymenoptera|COI-5P	87.8
OTU_1897;size=6;	Hymenoptera(63.7)	AUSCL1225-12|Hymenoptera|COI-5P	82.9
OTU_19;size=6009;	Hymenoptera(53.9)	ASPNA738-09|Formicidae|COI-5P|HM435142	88.6
OTU_190;size=801;	Hymenoptera(49.8)	GMBCC2372-15|Hymenoptera|COI-5P	93.9
OTU_1905;size=13;	Hymenoptera(60.0)	GBAH2167-06|Technomyrmex	84.1
OTU_191;size=474;	Hymenoptera(98.6)	GMMGT031-14|Hymenoptera|COI-5P	97.3
OTU_1922;size=6;	Hymenoptera(56.0)	ASPNA1797-11|Formicidae|COI-5P	89.6
OTU_1925;size=8;	Hymenoptera(99.3)	GBAHF071-13|Tetraponera	93.3
OTU_1931;size=5;	Hymenoptera(43.2)	GBMIN10137-12|Solenopsis	81.6
OTU_1941;size=5;	Hymenoptera(70.2)	ASPNA1324-10|Formicidae|COI-5P|HQ551019	89.3
OTU_1948;size=21;	Hymenoptera(53.9)	FSA1138-12|Hymenoptera|COI-5P	88.3
OTU_1969;size=5;	Hymenoptera(93.5)	ASAC049-07|Hymenoptera|COI-5P	84.8
OTU_20;size=5900;	Hymenoptera(98.4)	GBAH7014-10|Pachycondyla	91.1
OTU_2004;size=5;	Hymenoptera(53.9)	FSA627-12|Hymenoptera|COI-5P	88.8
OTU_2013;size=9;	Hymenoptera(57.9)	ASPNA712-09|Formicidae|COI-5P|HM435131	90.1
OTU_2026;size=5;	Hymenoptera(99.4)	GBAHF101-13|Polyrhachis	96
OTU_2027;size=5;	Hymenoptera(99.8)	WOMIC281-10|Microgastrinae|COI-5P|JN282285	100
OTU_205;size=505;	Hymenoptera(85.7)	GBAH11704-15|Nylanderia	100
OTU_208;size=552;	Hymenoptera(63.7)	ASPNA409-09|Formicidae|COI-5P|HM373292	88.1
OTU_209;size=444;	Hymenoptera(80.6)	ASANE557-10|Formicidae|COI-5P|JN287619	89.3
OTU_2092;size=5;	Hymenoptera(51.9)	NZHYM2059-13|Hymenoptera|COI-5P	91.1
OTU_2128;size=6;	Hymenoptera(40.8)	FSA298-12|Hymenoptera|COI-5P	86.6
OTU_2134;size=7;	Hymenoptera(99.3)	ASPNA2660-12|Hymenoptera|COI-5P	98.3
OTU_2152;size=10;	Hymenoptera(65.7)	JDWAM811-05|Anochetus	85.9
OTU_2188;size=10;	Hymenoptera(53.9)	GBAH2167-06|Technomyrmex	82.6
OTU_2210;size=4;	Hymenoptera(72.5)	ASPNA1097-10|Formicidae|COI-5P|HQ550826	85.9
OTU_224;size=838;	Hymenoptera(60.0)	ASANR249-09|Formicidae|COI-5P|GU711346	86.4
OTU_2262;size=8;	Hymenoptera(97.2)	ASPNA1330-10|Formicidae|COI-5P|HQ551024	92.3
OTU_2266;size=4;	Hymenoptera(61.8)	ASANR282-09|Formicidae|COI-5P|HM373190	85.4
OTU_2272;size=9;	Hymenoptera(88.1)	GBAHF327-13|Tapinoma	88.6
OTU_2284;size=4;	Hymenoptera(57.9)	GMBCC2372-15|Hymenoptera|COI-5P	93.6
OTU_2304;size=4;	Hymenoptera(99.6)	ASMA392-05|Technomyrmex	98.8
OTU_2305;size=4;	Hymenoptera(49.8)	ASLAM2180-12|Pheidole	79.9
OTU_2307;size=4;	Hymenoptera(56.0)	ASPNA2718-12|Hymenoptera|COI-5P	84.9
OTU_2311;size=8;	Hymenoptera(51.9)	ASPNA2562-12|Hymenoptera|COI-5P	80.6
OTU_2319;size=6;	Hymenoptera(53.9)	ASPNA1366-10|Formicidae|COI-5P|HQ551053	87.1
OTU_2322;size=6;	Hymenoptera(99.5)	CNCHW845-09|Microgastrinae|COI-5P|HM430642	100
OTU_2350;size=4;	Hymenoptera(98.8)	GBAHF076-13|Myrmica	91.3
OTU_2362;size=8;	Hymenoptera(70.2)	ICHFI1172-12|Hymenoptera|COI-5P	88.1
OTU_2366;size=4;	Hymenoptera(93.5)	ASMAI175-05|Pheidole	88.8
OTU_2374;size=4;	Hymenoptera(75.1)	ASANL376-10|Formicidae|COI-5P|HQ547206	87.8
OTU_2384;size=3;	Hymenoptera(98.8)	MAMTS101-14|Hymenoptera|COI-5P	97
OTU_2389;size=3;	Hymenoptera(70.2)	ASPNA536-09|Formicidae|COI-5P|HM373328	88.6

OTU_2392;size=3;	Hymenoptera(90.2)	ASPNA556-09|Formicidae|COI-5P|HM435079	88.6
OTU_2404;size=5;	Hymenoptera(51.9)	GMSAV1814-13|Hymenoptera|COI-5P	82.4
OTU_2430;size=3;	Hymenoptera(51.9)	GBMIN26719-13|Aphaenogaster	85.4
OTU_2511;size=3;	Hymenoptera(49.8)	BBHYA3111-12|Hymenoptera|COI-5P	83.6
OTU_252;size=365;	Hymenoptera(53.9)	ASPAL025-09|Hymenoptera|COI-5P	92.6
OTU_253;size=562;	Hymenoptera(61.8)	GBAHF076-13|Myrmica	99.5
OTU_2536;size=3;	Hymenoptera(99.6)	CNPPI204-12|Aprostocetus|COI-5P|KJ208035	88.4
**OTU_2567;size=3;***	**Hymenoptera(72.5)**	**HCNC039-09|Cymus**	**80.6**
**OTU_2573;size=3;***	**Hymenoptera(57.9)**	**COLLE348-09|Entomobryomorpha|COI-5P|HM397720**	**81.4**
OTU_2604;size=5;	Hymenoptera(28.8)	GMCHP007-14|Hymenoptera|COI-5P	93.4
OTU_2612;size=3;	Hymenoptera(60.0)	MAHYM468-10|Hymenoptera|COI-5P|JF866317	91.6
OTU_2627;size=3;	Hymenoptera(98.8)	ASPNA1482-10|Hymenoptera|COI-5P	88.6
OTU_2650;size=5;	Hymenoptera(47.7)	ASANZ114-10|Formicidae|COI-5P|HQ547537	82.9
OTU_267;size=359;	Hymenoptera(40.8)	ASPNA2849-12|Hymenoptera|COI-5P	85.1
OTU_2696;size=3;	Hymenoptera(47.7)	GBAHF101-13|Polyrhachis	92.3
OTU_270;size=371;	Hymenoptera(99.1)	GBMIN25372-13|Hymenoptera|COI-5P|JQ344567	100
OTU_2700;size=3;	Hymenoptera(99.5)	ASANG655-10|Hymenoptera|COI-5P	81.1
OTU_2729;size=3;	Hymenoptera(38.4)	GBAH11640-15|Nylanderia	88.5
OTU_2738;size=5;	Hymenoptera(72.5)	ASPAL025-09|Hymenoptera|COI-5P	85.9
OTU_2761;size=287;	Hymenoptera(88.1)	GBMIN25578-13|Hymenoptera|COI-5P|JQ345026	89.7
OTU_2858;size=2;	Hymenoptera(77.9)	ASANY176-10|Formicidae|COI-5P|HQ547326	84.9
OTU_293;size=497;	Hymenoptera(70.2)	GMCHE014-14|Hymenoptera|COI-5P	96.4
OTU_2934;size=2;	Hymenoptera(92.1)	SSBAF5460-13|Eulophidae|COI-5P|KR874027	90.3
OTU_2971;size=2;	Hymenoptera(77.9)	GBAH2167-06|Technomyrmex	83.9
**OTU_2986;size=2;***	**Hymenoptera(60.0)**	**MYFAB432-11|Lithobiomorpha|COI-5P|JN306635**	**76**
OTU_3;size=16375;	Hymenoptera(21.5)	GBAH2166-06|Odontoponera	92.3
OTU_3015;size=2;	Hymenoptera(99.2)	GBAHF084-13|Camponotus	100
**OTU_3017;size=2;***	**Hymenoptera(99.8)**	**LNOUB700-10|Lepidoptera|COI-5P|HQ988948**	**77.4**
OTU_304;size=233;	Hymenoptera(43.2)	GMBCD243-15|Hymenoptera|COI-5P	94.4
OTU_3040;size=2;	Hymenoptera(90.2)	ASPNA2424-12|Hymenoptera|COI-5P	83.6
OTU_3053;size=2;	Hymenoptera(47.7)	ASPNA1451-10|Hymenoptera|COI-5P	99.3
OTU_307;size=1389;	Hymenoptera(53.9)	GBAH6996-10|Pachycondyla	91.1
OTU_3084;size=2;	Hymenoptera(94.8)	ASPNA2083-11|Formicidae|COI-5P	88.1
OTU_3089;size=2;	Hymenoptera(45.5)	ASPNA2283-11|Hymenoptera|COI-5P	89.3
OTU_3099;size=2;	Hymenoptera(63.7)	ASANG664-10|Formicidae|COI-5P|JF862924	90.6
OTU_3100;size=2;	Hymenoptera(92.1)	PHFLO124-10|Hymenoptera|COI-5P|JN307266	86.1
OTU_3104;size=2;	Hymenoptera(60.0)	ASAMF130-06|Hymenoptera|COI-5P	98.5
OTU_3114;size=2;	Hymenoptera(99.4)	ASSPQ024-08|Baryscapus	92.4
OTU_3122;size=2;	Hymenoptera(98.6)	ASANX177-10|Formicidae|COI-5P|HM880834	84.4
OTU_318;size=677;	Hymenoptera(53.9)	GMBCD243-15|Hymenoptera|COI-5P	92.3
OTU_3181;size=2;	Hymenoptera(92.1)	GMBCC2372-15|Hymenoptera|COI-5P	94.8
OTU_3185;size=2;	Hymenoptera(60.0)	ASANJ593-11|Formicidae|COI-5P	83.4
OTU_3189;size=2;	Hymenoptera(61.8)	MAMTN097-13|Hymenoptera|COI-5P	86.4

OTU_3192;size=2;	Hymenoptera(40.8)	GMBCC2372-15|Hymenoptera|COI-5P	92.1
OTU_3203;size=2;	Hymenoptera(45.5)	CNCHW731-09|Apanteles|COI-5P|HM430546	95.3
OTU_3237;size=2;	Hymenoptera(99.3)	ASANI781-11|Hymenoptera|COI-5P	84.4
OTU_3286;size=2;	Hymenoptera(60.0)	ASPAL025-09|Hymenoptera|COI-5P	92.6
OTU_3298;size=2;	Hymenoptera(57.9)	INAOB012-12|Aphaenogaster	80.1
OTU_3300;size=2;	Hymenoptera(57.9)	MAHYM391-10|Hymenoptera|COI-5P	89.7
OTU_3309;size=2;	Hymenoptera(67.8)	ASANS508-10|Formicidae|COI-5P|HM418723	84.6
OTU_3320;size=2;	Hymenoptera(47.7)	CNGRL255-13|Encyrtidae|COI-5P|KR789888	87.2
OTU_3332;size=2;	Hymenoptera(61.8)	ASPNA2242-11|Hymenoptera|COI-5P	85.6
OTU_3344;size=2;	Hymenoptera(40.8)	CNSLS095-13|Spathius|COI-5P|KR797084	91.3
OTU_335;size=199;	Hymenoptera(72.5)	FSA1049-12|Hymenoptera|COI-5P	85.9
OTU_3377;size=2;	Hymenoptera(56.0)	ASPNA409-09|Formicidae|COI-5P|HM373292	89.1
OTU_3378;size=2;	Hymenoptera(60.0)	INAOB016-12|Aphaenogaster	76.7
OTU_3421;size=2;	Hymenoptera(33.3)	ETK026-11|Formicidae|COI-5P	85.9
OTU_3436;size=2;	Hymenoptera(61.8)	ASANO837-09|Pheidole	85.9
OTU_3450;size=2;	Hymenoptera(80.6)	GMMGT2846-14|Hymenoptera|COI-5P	85.4
OTU_3488;size=2;	Hymenoptera(28.8)	BCHYM4407-14|Hymenoptera|COI-5P	90.6
OTU_3512;size=2;	Hymenoptera(80.6)	HYCNI1541-12|Hymenoptera|COI-5P	87.4
OTU_3521;size=2;	Hymenoptera(61.8)	GBAH5515-09|Messor	85.1
OTU_3545;size=2;	Hymenoptera(51.9)	GBAHF077-13|Cerapachys	87.1
OTU_355;size=227;	Hymenoptera(63.7)	GMCHP006-14|Hymenoptera|COI-5P	90
OTU_3558;size=2;	Hymenoptera(63.7)	GBAHF075-13|Paraparatrechina	90.6
OTU_356;size=199;	Hymenoptera(90.2)	GBAHF075-13|Paraparatrechina	99.3
**OTU_3567;size=4;***	**Hymenoptera(99.6)**	**GBMIN26777-13|Orius**	**84.4**
OTU_3577;size=2;	Hymenoptera(56.0)	GBAH6996-10|Pachycondyla	87.8
OTU_3581;size=2;	Hymenoptera(70.2)	GBAHF077-13|Cerapachys	86.8
OTU_3586;size=2;	Hymenoptera(57.9)	GMCHP006-14|Hymenoptera|COI-5P	91.5
OTU_3589;size=2;	Hymenoptera(72.5)	ASPNA2161-11|Formicidae|COI-5P	84.9
OTU_3593;size=2;	Hymenoptera(47.7)	GBAHF325-13|Technomyrmex	90.8
OTU_3612;size=2;	Hymenoptera(94.8)	GMCHP006-14|Hymenoptera|COI-5P	92.4
OTU_364;size=255;	Hymenoptera(94.8)	GMBCC2372-15|Hymenoptera|COI-5P	89.6
OTU_368;size=194;	Hymenoptera(51.9)	GMCHE014-14|Hymenoptera|COI-5P	95.5
OTU_375;size=159;	Hymenoptera(92.1)	GBAHF332-13|Dolichoderus	88.8
OTU_381;size=149;	Hymenoptera(98.0)	GBMIN25578-13|Hymenoptera|COI-5P|JQ345026	90.3
OTU_398;size=220;	Hymenoptera(57.9)	GMMGN1711-14|Hymenoptera|COI-5P	88.4
OTU_399;size=210;	Hymenoptera(63.7)	GMMGS722-14|Hymenoptera|COI-5P	91
OTU_405;size=129;	Hymenoptera(80.6)	GBAHF332-13|Dolichoderus	85.1
OTU_411;size=285;	Hymenoptera(51.9)	GBAH11702-15|Nylanderia	99.8
OTU_421;size=121;	Hymenoptera(67.8)	ASPAL025-09|Hymenoptera|COI-5P	90.3
OTU_428;size=112;	Hymenoptera(80.6)	NOFOR028-13|Hymenoptera|COI-5P	84.9
OTU_445;size=153;	Hymenoptera(33.3)	ASPAL025-09|Hymenoptera|COI-5P	89.6
OTU_455;size=96;	Hymenoptera(65.7)	ASPAN281-10|Formicidae|COI-5P|JF863830	83.6
OTU_457;size=92;	Hymenoptera(80.6)	GMBCD243-15|Hymenoptera|COI-5P	94.1

OTU_46;size=2956;	Hymenoptera(77.9)	GBAH6998-10|Pachycondyla	90.6
OTU_462;size=266;	Hymenoptera(98.0)	ASPNA1084-10|Formicidae|COI-5P|HQ550817	83.4
OTU_466;size=108;	Hymenoptera(38.4)	BASYM1197-11|Hymenoptera|COI-5P|KC972652	79.2
OTU_469;size=171;	Hymenoptera(57.9)	GMNZE548-14|Hymenoptera|COI-5P	92.1
OTU_475;size=122;	Hymenoptera(94.8)	MGABB398-10|Formicidae|COI-5P|HM893002	91.1
OTU_477;size=138;	Hymenoptera(98.0)	GBAH0134-06|Oecophylla	100
OTU_478;size=230;	Hymenoptera(99.9)	ASANI350-11|Formicidae|COI-5P	84.9
**OTU_479;size=89;***	**Hymenoptera(47.7)**	**GBDP4990-08|Zaprionus**	**97**
OTU_48;size=3686;	Hymenoptera(60.0)	ASPAL025-09|Hymenoptera|COI-5P	89.3
OTU_494;size=160;	Hymenoptera(90.2)	GBAH11704-15|Nylanderia	94
OTU_5;size=17242;	Hymenoptera(33.3)	GMMGO010-14|Hymenoptera|COI-5P	88.6
OTU_505;size=105;	Hymenoptera(72.5)	ASPNA1482-10|Hymenoptera|COI-5P	89.3
OTU_518;size=111;	Hymenoptera(92.1)	MAHYM468-10|Hymenoptera|COI-5P|JF866317	89.3
OTU_53;size=4237;	Hymenoptera(98.2)	GBAH6996-10|Pachycondyla	91.6
OTU_551;size=72;	Hymenoptera(99.3)	ASANX117-10|Formicidae|COI-5P|HM419386	86.6
OTU_554;size=91;	Hymenoptera(51.9)	GBAH5187-09|Neodiprion	81.5
OTU_565;size=62;	Hymenoptera(51.9)	ASLAM479-11|Pachycondyla	82.6
OTU_599;size=57;	Hymenoptera(63.7)	ASANY108-10|Hymenoptera|COI-5P	87.6
OTU_603;size=63;	Hymenoptera(80.6)	GMBCF3807-15|Hymenoptera|COI-5P	89.3
OTU_61;size=2745;	Hymenoptera(99.1)	GBAH7010-10|Pachycondyla	91.8
OTU_637;size=54;	Hymenoptera(51.9)	GBMIN26678-13|Camponotus	82.9
OTU_66;size=2071;	Hymenoptera(94.8)	ASANA493-06|Hymenoptera|COI-5P	90.8
OTU_684;size=54;	Hymenoptera(60.0)	GBMIN11072-12|Nylanderia	78.2
OTU_685;size=46;	Hymenoptera(67.8)	GBAHF117-13|Anoplolepis|COI-5P|JQ681050	98.5
OTU_693;size=54;	Hymenoptera(35.8)	ASANQ417-09|Formicidae|COI-5P|GU711066	78.7
OTU_694;size=40;	Hymenoptera(88.1)	GBAHF327-13|Tapinoma	89.8
OTU_707;size=44;	Hymenoptera(51.9)	GBAH11583-15|Monomorium	82.9
OTU_73;size=2042;	Hymenoptera(61.8)	GMCHP006-14|Hymenoptera|COI-5P	91.2
OTU_740;size=55;	Hymenoptera(47.7)	FSA184-11|Formicidae|COI-5P	85.1
OTU_750;size=45;	Hymenoptera(60.0)	ASANY511-10|Formicidae|COI-5P|KF200838	85.9
OTU_758;size=40;	Hymenoptera(99.4)	ASPAL044-09|Hymenoptera|COI-5P	97.5
OTU_769;size=34;	Hymenoptera(47.7)	GBAH11542-15|Monomorium	84.6
OTU_773;size=31;	Hymenoptera(75.1)	ICHFI295-12|Hymenoptera|COI-5P	84.6
OTU_779;size=40;	Hymenoptera(45.5)	ASPNA516-09|Formicidae|COI-5P|HM373323	86.1
OTU_8;size=10162;	Hymenoptera(99.2)	GBAHF327-13|Tapinoma	93.5
OTU_805;size=31;	Hymenoptera(98.8)	ASPNA741-09|Formicidae|COI-5P|HQ927082	97.3
OTU_807;size=32;	Hymenoptera(63.7)	FSA1848-13|Hymenoptera|COI-5P	89.1
OTU_822;size=69;	Hymenoptera(98.0)	ASANA717-06|Hymenoptera|COI-5P	84.6
OTU_835;size=39;	Hymenoptera(80.6)	ASLAM140-08|Pheidole	86.8
OTU_841;size=26;	Hymenoptera(35.8)	GBMIN25809-13|Hymenoptera|COI-5P|JQ344560	100
OTU_844;size=26;	Hymenoptera(99.7)	GBAHF325-13|Technomyrmex	100
OTU_86;size=2421;	Hymenoptera(98.7)	GBAH6996-10|Pachycondyla	90.6
OTU_864;size=25;	Hymenoptera(98.0)	GMMGD269-14|Hymenoptera|COI-5P	92.8

OTU_877;size=31;	Hymenoptera(98.8)	GBAH6996-10|Pachycondyla	92.1
OTU_88;size=1656;	Hymenoptera(72.5)	ASANY107-10|Formicidae|COI-5P|HQ547310	87.1
OTU_89;size=3148;	Hymenoptera(63.7)	GMCHP006-14|Hymenoptera|COI-5P	89.7
OTU_934;size=21;	Hymenoptera(97.2)	GBAH7019-10|Pachycondyla	90.6
OTU_935;size=21;	Hymenoptera(60.0)	ASPNA2402-12|Hymenoptera|COI-5P	87.6
OTU_949;size=21;	Hymenoptera(35.8)	KHYME5150-13|Hymenoptera|COI-5P	78.5
OTU_957;size=41;	Hymenoptera(47.7)	MAMTD487-12|Hymenoptera|COI-5P	84.1
OTU_960;size=21;	Hymenoptera(98.9)	GBAHF102-13|Polyrhachis	94
OTU_976;size=52;	Hymenoptera(63.7)	ASPNA738-09|Formicidae|COI-5P|HM435142	89.7
OTU_98;size=2148;	Hymenoptera(98.2)	GBAHF336-13|Dolichoderus	90.6
OTU_984;size=38;	Hymenoptera(63.7)	ASPNA738-09|Formicidae|COI-5P|HM435142	89.9
OTU_990;size=22;	Hymenoptera(72.5)	GBAHF439-14|Pheidole	87.8

Table S5 MOTU richness differences between mono- and polyculture tea, and between terraced and unterraced tea for all MOTUs combined and MOTUs coarsely identified to Orders. Differences are based on Kruskal-Wallis rank sum test and Mann-Whitney U test with bonferroni correction.

MOTUs	X^2	P	Pairwise comparisons	Z	P
All	2.122	0.15	Poly-/Monoculture	1.456	0.072
	0.011	0.92	Terraced/Unterraced	-0.106	0.457
Arachnida	0.592	0.44	Poly-/Monoculture	0.76	0.220
	0.581	0.45	Terraced/Unterraced	0.762	0.222
Blattodea	0.007	0.93	Poly-/Monoculture	0.088	0.464
	1.247	0.26	Terraced/Unterraced	1.116	0.132
Coleoptera	2.674	0.1	Poly-/Monoculture	1.635	0.051
	1.130	0.29	Terraced/Unterraced	-1.063	0.143
Diptera	1.425	0.23	Poly-/Monoculture	1.193	0.116
	0.045	0.83	Terraced/Unterraced	0.212	0.415
Hemiptera	3.649	0.06	Poly-/Monoculture	1.910	0.028
	1.929	0.16	Terraced/Unterraced	-1.389	0.082
Hymenoptera	3.287	0.07	Poly-/Monoculture	1.813	0.034
	0.138	0.71	Terraced/Unterraced	-0.372	0.354
Chilopoda	1.706	0.19	Poly-/Monoculture	1.306	0.095
	0.660	0.42	Terraced/Unterraced	-0.812	0.208
Orthoptera	0.017	0.89	Poly-/Monoculture	0.132	0.447
	1.131	0.29	Terraced/Unterraced	-1.063	0.143

Table S6 Taxonomic identification levels for eight arthropod groups

		% identified to			
	No. of MOTUs	Class	Order	Family	Genus
Blattodea	319	-	75.9	-	24.1
Coleoptera	731		52.4	2.1	45.5
Diptera	517		52.6	5.6	41.8
Hemiptera	246		63.8	2.1	34.1
Hymenoptera	294		46.9	24.8	28.2
Orthoptera	227	-	35.7	-	64.3
Arachnida	88	5.7	62.5	10.2	21.6
Chilopoda	147	1.4	45.6	1.4	51.7

Land use types and characteristics

1. *Native vegetation (henceforth Forest)*: The tropical vegetation of XSBN is classified into four forest types; tropical seasonal rainforests, tropical montane rainforests, tropical seasonal moist forests and tropical montane evergreen broad-leaf forests [1]. These forest types differ from each other in terms of spatial distribution, elevation, species diversity, physiognomy, vegetation structure and composition [1].
2. *Rubber plantations (henceforth Rubber)*: The most substantial land use change in XSBN is the conversion of native forests to rubber. Forests (mostly below 900 m a.s.l.) are clear-cut and burnt. Terraces (~ 1 m high and ~ 2.5 m wide) are cut into the ground and young rubber seedlings are planted on the terraces [2]. Management also includes understory cover control using herbicides or manual cutting, application of synthetic fertilizers (Nitrogen-Phosphorus-Potassium [N-P-K] complex), pest control and latex collection [2]. All these, except latex collection, take place once or twice a year and constitute ecosystem disturbance. Latex is collected every second day between March /April and October / November each year.
3. *Tea plantations (henceforth Tea)*: Unlike rubber where terrace benches are always constructed, tea is sometimes planted in the forest understory or together with other trees and/or crops [3]. Until recently, only large-scale commercial tea production used ‘contour planting’, where tea bushes are planted in lines along elevational isoclines, usually on clear-cut slopes. Pesticide and fertilizer use are a common practice in most tea plantations.

Land use change and environment variables

To evaluate the factors determining arthropod richness, rarity and composition patterns within natural and human-modified habitats, we assessed land use and environmental variables as follows:

1. Habitat: categorized into three types ordered according to disturbance level (e.g. forest [0], tea [1] and rubber [2]).
2. Canopy openness: extracted from nine hemispheric (true-color fisheye) photographs taken at each site, with canopy openness determined from the photographs using the Gap Light Analyzer (GLA) version 2.0 software [4], and averaged.
3. Leaf litter thickness: measured with a ruler at three points within each of the nine sampling quadrats (27 measurements per site) and averaged
4. Leaf litter quality: Total Carbon (TC), total Nitrogen (TN), total Phosphorus (TP), total Potassium (TK), tannin content, coarse fiber content and (acid-digestible) lignin content were analyzed from composite leaf litter samples of each site at the Xishuangbanna Tropical Botanical Garden (XTBG) Biogeochemistry Laboratory. TC and TN concentrations (g/Kg) were determined using the vario Max CN Elemental Analyzer (elementar Analysensysteme GmbH, Germany). TP and TK concentrations (g/Kg) were determined using the ICP-AES (iCAP 6300, Thermo Fisher Scientific Inc, USA) method. Tannin (%) and lignin contents (%) were determined using a UV-Visible Spectrophotometer (UV-2450, Shimadzu, Japan). Coarse fiber content (%) was determined using the automated fibertec method (Fibertec ™ 2010, Foss Analytical; Denmark).

5. Environment: elevation recorded with a hand-held GPS (GPSMAP® 62s | Garmin); slope measurements were taken at three of the quadrats for each site with a clinometer (Suunto Clinometer 802575) and averaged; topography, a factor with three levels as valley (lower quarter of a hillside =0), slope (the intermediate slope positions =1), and ridge (upper quarter of a hillside =2).

References

1. 1 Zhu, H., Cao, M. and Hu, H. (2006), Geological History, Flora, and Vegetation of Xishuangbanna, Southern Yunnan, China. Biotropica, 38: 310–317. doi: 10.1111/j.1744-7429.2006.00147.x
2. de Blécourt M, Brumme R, Xu J, Corre MD, Veldkamp E (2013). Soil Carbon Stocks Decrease following Conversion of Secondary Forests to Rubber (*Hevea brasiliensis*) Plantations. PLoS ONE 8(7): e69357. doi:10.1371/journal.pone.0069357
3. Liu SJ, Li JP, Sui Y, Schaefer DA, Alele PO, Chen J, Yang XD (2015) Spider foraging strategies dominate pest suppression in organic tea plantations. Biol Control doi: 10.1007/s10526-015-9691-2
4. Frazer, G.W., Canham, C.D., and Lertzman, K.P. (1999). Gap Light Analyzer (GLA), Version 2.0: Imaging software to extract canopy structure and gap light transmission indices from true-color fisheye photographs, user's manual and program documentation. Copyright © 1999: Simon Fraser University, Burnaby, British Columbia, and the Institute of Ecosystem Studies, Millbrook, New York.
